# Pan-Cancer Analysis of IGF-1 and IGF-1R as Potential Prognostic Biomarkers and Immunotherapy Targets

**DOI:** 10.3389/fonc.2021.755341

**Published:** 2021-11-05

**Authors:** Yinqi Zhang, Chengqi Gao, Fei Cao, Ying Wu, Shuanggang Chen, Xue Han, Jingqin Mo, Zhiyu Qiu, Weijun Fan, Penghui Zhou, Lujun Shen

**Affiliations:** ^1^ Zhongshan School of Medicine, Sun Yat-Sen University, Guangzhou, China; ^2^ Department of Minimally Invasive Interventional Therapy, Sun Yat-Sen University Cancer Center, Guangzhou, China; ^3^ State Key Laboratory of Oncology in South China, Collaborative Innovation Center of Cancer Medicine, Sun Yat-Sen University, Guangzhou, China; ^4^ Department of Oncology, Yuebei People’s Hospital, Shantou University Medical College, Shaoguan, China

**Keywords:** pan-cancer, IGF-1, IGF-1R, prognostic biomarker, immunity

## Abstract

**Aim:**

Insulin-like growth factor-1 receptor (IGF-1R) is one of the main members of the tyrosine protein kinase receptor family. This receptor binds insulin-like growth factor-1 (IGF-1) with a high affinity. IGF-1 is a member of a family of proteins involved in mediating growth and development. However, the correlations of IGF-1 and IGF-1R to prognosis and tumor-infiltrating lymphocytes in different cancers remain unclear.

**Method:**

This research comprehensively analyzed the expression pattern of IGF-1 and IGF-1R and the influence of IGF-1 and IGF-1R on clinical significance in prognosis prediction among 33 types of malignancies using The Cancer Genome Atlas (TCGA) and the Cancer Cell Line Encyclopedia (CCLE) databases. The correlation between IGF-1, IGF-1R, and cancer immunity was explored.

**Results:**

IGF-1 and IGF-1R displayed inconsistent gene expression levels among diverse cancer cell lines. Typically, high expression level of IGF-1 and IGF-1R was detected in most malignant tumors. High expression of IGF-1 was closely bound up with the unfavorable overall survival (OS) for patients in BLCA, CHOL, and LAML upon Cox and Kaplan-Meier analyses. While high expression of IGF-1R was closely bound up with the unfavorable overall survival (OS) for patients in BLCA, LIHC, and LUAD. Furthermore, high expression level of IGF-1 and IGF-1R were closely connected with high degrees of tumor infiltrates, including CD4+ T cell, dendritic cells, and macrophages. In addition, we found that IGF-1 was commonly positively correlated with the expression of gene markers including LAIR1, ICOS, CD40LG, CTLA4, CD48, CD28, CD200R1, HAVCR2, and CD86. Whereas, IGF-1R was commonly positively correlated with the expression of gene markers including NRP1 and CD276. More importantly, IGF-1 and IGF-1R expression were correlated with tumor mutation burden (TMB), microsatellite instability (MSI), mismatch repair (MMR), and DNA methyltransferase (DNMT) of different types of cancers.

**Conclusions:**

The impact of high IGF-1 and IGF-1R on prognosis and immune infiltrates differs across cancer types. Anti-IGF-1R therapy may inhibit tumor growth and contribute to immunotherapy in LIHC and KIRC.

## Introduction

Insulin-like growth factor 1 receptor (IGF-1R), one of the main members of tyrosine protein kinase receptor family, plays an important role in maintaining the malignant phenotype and tumor anti-apoptosis. Insulin-like growth factor-1 (IGF-1), ligand of IGF-1R, is a kind of growth hormone mainly synthesized in liver. Population studies provide substantial direct and circumstantial evidence that cancer risk and cancer prognosis are influenced by IGF-1 and insulin levels (1). The overexpression of IGF-1 and its receptor IGF-1R have been implicated in carcinogenesis and are also considered risk factors for the progression of diverse human cancers (2–4). On the other hand, studies have proved that anti-IGF-1R monoclonal antibody has potential therapeutic value in diverse cancers (5–7). Researchers have also found that the differentiation of *ex vivo*-expanded CD34+ cells through manipulation of RAS/MAPK, IGF-1R, and TGF-β signaling pathways is an efficient approach for generating functional NK cells that can be used for cancer immunotherapy (8). However, the correlations of IGF-1 and IGF-1R to prognosis and tumor-infiltrating lymphocytes in different cancers remain unclear. Since cancer is a leading cause of death worldwide, and the low efficacy of many existing therapies is a major clinical challenge, it is essential to understand the prognostic and immunological impact of IGF-1 and IGF-1R among cancer types comprehensively in order to develop novel immunotherapies.

Molecular-level pan-cancer analyses have provided insights into the common features and heterogeneity of various human malignancies. Since the establishment of The Cancer Genome Atlas based on various human cancer samples and normal tissues at epigenomic, genomic, proteomic, and transcriptomic levels, diverse cancer samples are offered so that deeper pan-cancer analysis could be conducted (9). Therefore, we conducted a pan-cancer analysis taking advantage of its large datasets. The analysis aimed to (a) describe the expression of IGF-1 and IGF-1R among different cancer types; (b) assess the prognostic values of IGF-1 and IGF-1R among varied tumors; and (c) evaluate the associations between IGF-1/IGF-1R and tumor immunity features including intratumoral immune infiltrates, checkpoint markers, tumor mutation burden (TMB), and microsatellite instability (MSI), which have been identified as biomarkers for predicting response to immune checkpoint inhibitor treatment (10).

## Methods

### Patient Datasets and Processing

The Cancer Genome Atlas (TCGA), a milestone of the cancer genomics project, characterizes thousands of primary cancer samples and matched adjacent noncarcinoma samples from 33 types of cancers. In this study, the TCGA level 3 RNA sequencing processed data and the corresponding clinical annotations were acquired using the UCSC cancer genome browser (https://tcga.xenahubs.net, accessed May 2020). The Cancer Cell Line Encyclopedia (CCLE) public project is established through the comprehensive characterization of tremendous human tumor models at both genetic and pharmacological levels (https://portals.broadinstitute.org/ccle). To examine the differential gene expression in cancers at a larger a scale, the CCLE database containing the RNA-sequencing datasets for over 1,000 cell lines (https://portals.broadinstitute.org/ccle) was used in this study. The approval from the Ethics Committee was exempted as only the open-access data were used.

### IGF-1/IGF-1R Differential Expression and Survival-Associated Cancers

To compare the gene expression levels between cancer and adjacent noncarcinoma samples, data regarding the gene expression profiles of IGF-1/IGF-1R were extracted from the 33 cancer types in TCGA to from an expression matrix. It is thereafter merged with corresponding clinical information by patient ID. Univariate Cox model was applied in calculating the associations between gene expression levels and patient survival among 33 cancer types, and a difference of *p* < 0.05 for IGF-1 and IGF-1R in a specific cancer indicated statistical significance. The survival-associated forest plot is also made. Moreover, the Kaplan-Meier (KM) analysis by log-rank test was conducted to compare the overall survival (OS) and disease-specific survival (DSS) for TCGA cancer patients stratified based on the median gene expression level of IGF-1/IGF-1R.

### IGF-1/IGF-1R and Tumor Immune Microenvironment

The tumor-infiltrating immunocyte levels among different types of cancers were estimated by Tumor Immune Estimation Resource (TIMER, https://cistrome.shinyapps.io/timer/) (11) and CIBERSORT (12) based on related gene expression data, through deconvolution statistical method. The relationships between each immune infiltrate among 33 cancer types and IGF-1/IGF-1R expression were analyzed.

Estimation of STromal and Immune cells in MAlignant Tumor tissues using Expression data (ESTIMATE) is used to predict tumor purity and the infiltrating stromal cells/immunocytes in tumor tissue based on gene expression profiles (13). The ESTIMATE algorithm produces three scores based on the gene set enrichment analysis (GSEA) of single samples, including stromal score, which determines stromal cells in tumor tissues and immune score, which stands for the immunocyte infiltration level in tumor tissues. In this study, ESTIMATE algorithm is used to estimate the stromal and immune scores in tumor tissues according to corresponding transcriptional data and calculated the correlations of these scores with the expression of IGF-1/IGF-1R.

The relationships between the expression level of IGF-1/IGF-1R and the gene markers in tumor infiltrating immunocytes selected with reference to previous research were further conducted (14, 15). Correlation analysis was conducted to generate the estimated statistical significance and Spearman’s correlation coefficient. The somatic mutation data of all TCGA patients were downloaded (https://tcga.xenahubs.net) in order to calculate TMB scores and MSI scores and explore the correlation of IGF1/IGF-1R with MMR genes and DNMT.

### Gene Set Enrichment Analysis

GSEA were performed on IGF-1 and IGF-1R to understand the interrelated biological functions and pathways of IGF-1/IGF-1R. The molecular signature Database (MSigDB) H (hallmark gene sets) collection of chemical and genetic perturbations and KEGG subsets of canonical pathways and cancer modules were employed, and the analysis was completed on Sangerbox (http://sangerbox.com/). Normalized enrichment scores (NES) were used to show GSEA results, accounting for the size and degree to which a gene set is overrepresented at the top or bottom of the ranked list of genes (nominal *p*-value <0.05 and FDR ≤ 0.25). Bioconductor (http://bioconductor.org/) and R software (http:///www.r-project.org/) were used to visualize the enrichment maps of results.

### Statistical Analysis

In the present work, the clinical survival types, including OS and disease-specific survival (DSS), were selected for analysis. Generally, OS is deemed as the duration from the date of diagnosis to the date of death due to any course, DSS is considered disease progression or death due to the disease.

Wilcox log rank test was adopted to determine the presence or absence of a markedly increased sum of gene expression z-scores in cancer tissues compared with adjacent normal tissues. Meanwhile, Kruskal-Wallis test was employed to compare the difference in the expression of IGF-1 and IGF-1R. Survival was analyzed by the KM curves, log-rank test, and the Cox proportional hazard regression model. Spearman’ test was utilized for correlation analysis. The R language (version 3.6.0; R Foundation) was used for all analyses. A two-sided difference of *p* < 0.05 indicated statistical significance.

## Results

### Pan-Cancer Expression Landscape of IGF-1 and IGF-1R

**Figure 1:**
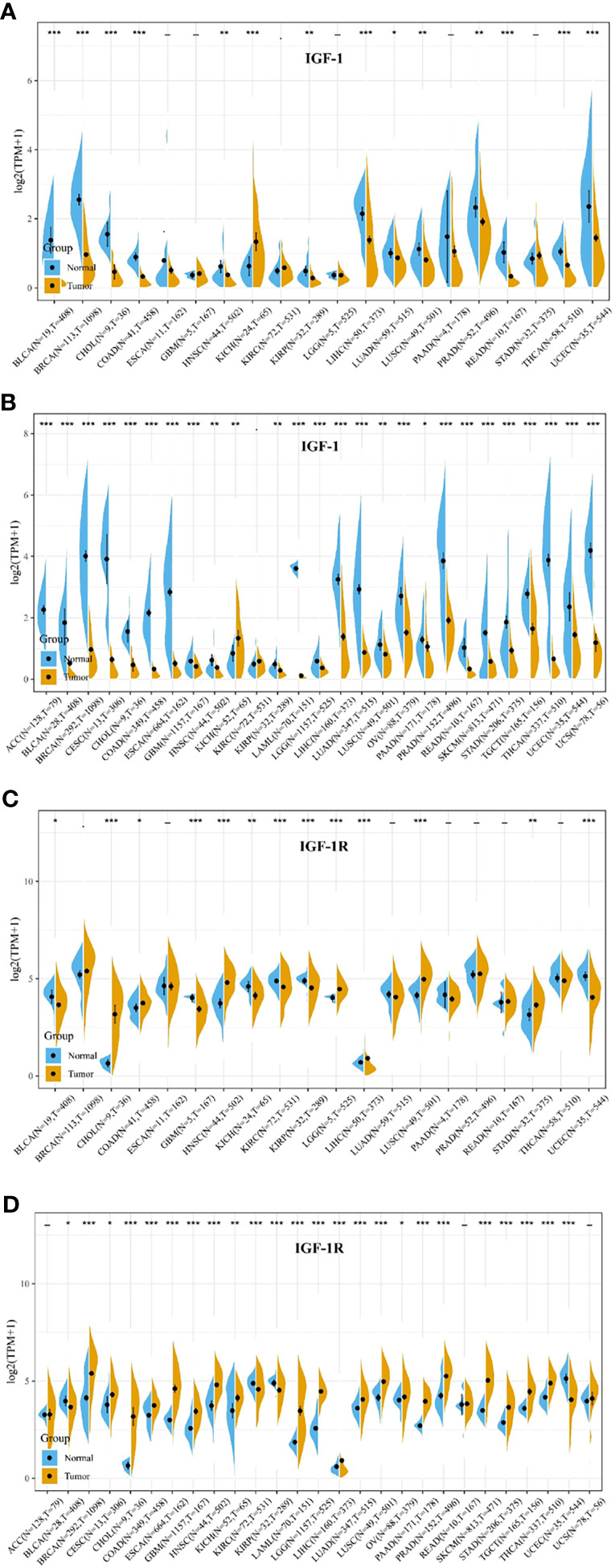
IGF-1/IGF-1R expression levels in different types of human cancers. The expression levels of IGF-1 between tumor and normal tissues were compared in 20 cancer types based on the TCGA database **(A)** and 27 cancer types based on the integrated database from TCGA and GTEx datasets **(B)**. The expression levels of IGF-1R between tumor and normal tissues were compared in 20 cancer types based on the TCGA database **(C)** and 27 cancer types based on the integrated database from TCGA and GTEx datasets **(D)**. Consistent high expression level of IGF-1 could be seen in normal tissues than most types of tumor based on both comparisons, and significant decreased expression of IGF-1 could be seen in tumor samples including ACC, BLCA, BRCA, CESC, CHOL, COAD, ESCA, LAML, LGG, LIHC, LUAD, OV, PRAD, READ, SKCM, STAD, TGCT, THCA, UCEC, and UCS based on the integrated database. Consistent high expression level of IGF-1R could be seen in most types of tumor than normal tissue based on the both comparisons, and significant increased expression of IGF-1R could be seen in tumor samples including BRCA, CHOL, COAD, ESCA, GBM, HNSC, LAML, LGG, LIHC, LUAD, LUSC, PAAD, PRAD, SKCM, STAD, TGCT, and THCA based on the integrated database. “*, **, ***” means p < 0.05, p < 0.01 and p < 0.001, respectively.

Comparison of expression of IGF-1/IGF-1R between normal and tumor samples across TCGA cancer types and the combined datasets based on integrated database of GTEx and TCGA datasets were conducted and shown in **Figure 1**. Consistent high expression level of IGF-1 could be seen in normal tissues than most types of tumor based on both comparisons, and significant decreased expression of IGF-1 could be seen in tumor samples including ACC, BLCA, BRCA, CESC, CHOL, COAD, ESCA, LAML. LGG, LIHC, LUAD, OV, PRAD, READ, SKCM, STAD, TGCT, THCA, UCEC, and UCS based on the integrated database. Whereas, high expression level of IGF-1R could be seen in most types of tumors than normal tissue based on both comparisons, and significant increased expression of IGF-1R could be seen in tumor samples including BRCA, CHOL, COAD, ESCA, GBM, HNSC, LAML, LGG, LIHC, LUAD, LUSC, PAAD, PRAD, SKCM, STAD, TGCT, and THCA based on the integrated database. Patients with different tumor stage and gender did not differ in the expression of IGF-1/IGF-1R in tumor samples.

### Correlation of IGF-1/IGF-1R Expression Level and Overall Survival of Cancer Patients

**Figure 2:**
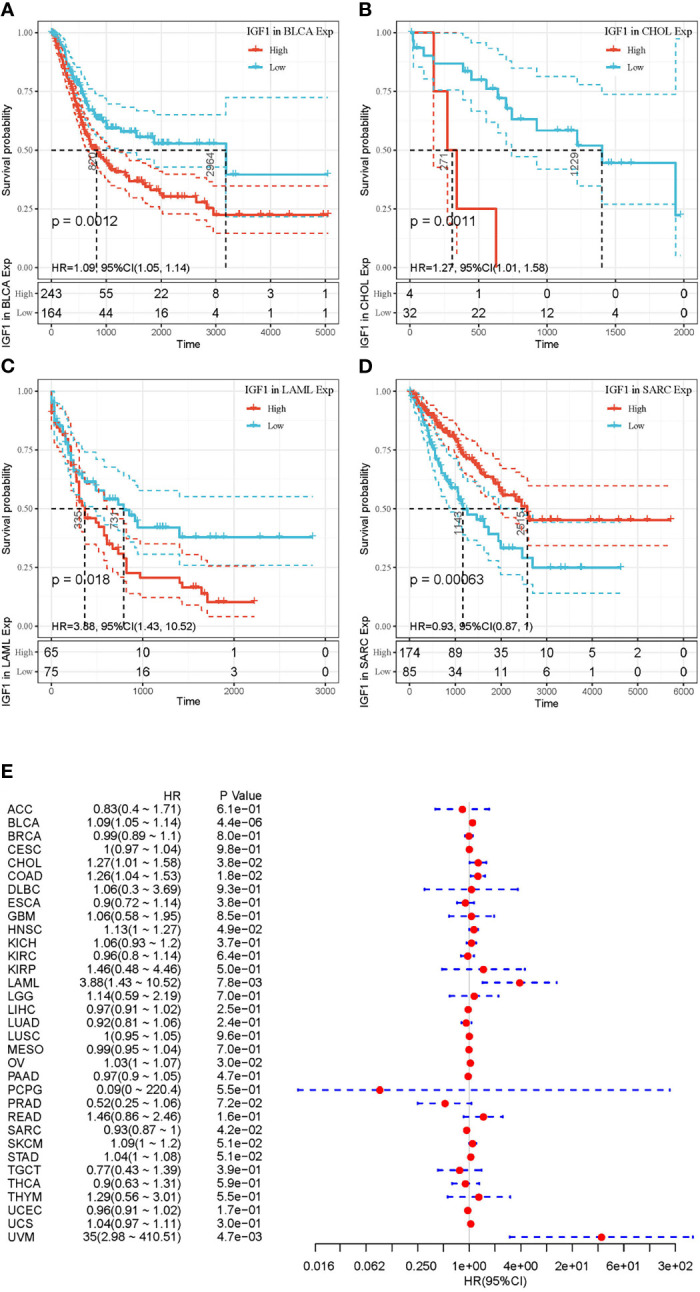
Selected Kaplan-Meier plots and forest plot comparing the high and low expressions of IGF-1 on overall survival across different cancers. **(A–C)** Kaplan-Meier method showed high expression of IGF-1 correlated with unfavorable prognosis in BLCA, CHOL, and LAML. **(D)** Kaplan-Meier method showed high expression of IGF-1 correlated with favorable prognosis in SARC. **(E)** Forest plot displaying the impact of high expression of IGF-1 on OS across 33 cancer types using Cox regression model. Confidence level is shown in dashed lines.

**Figure 3:**
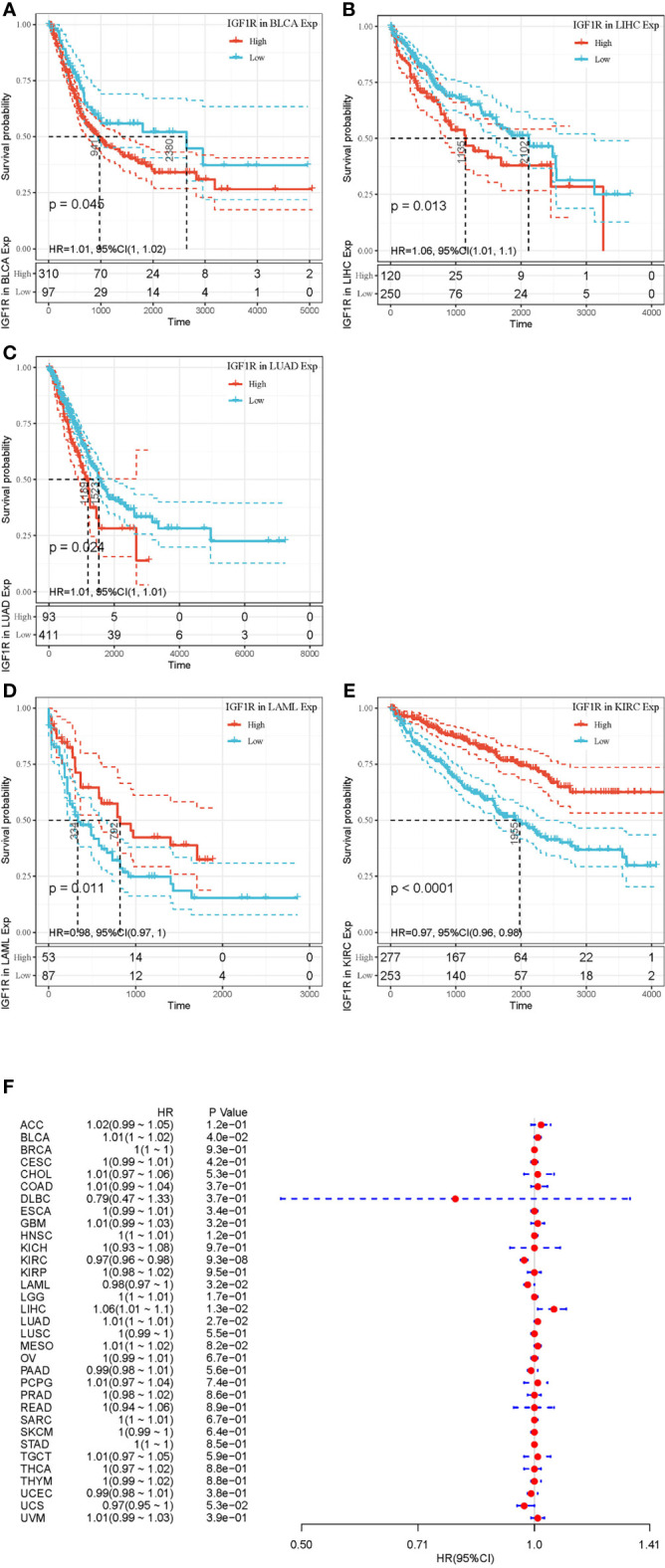
Selected Kaplan-Meier plots and forest plot comparing the high and low expressions of IGF-1R on overall survival across different cancers. **(A–C)** Kaplan-Meier method showed high expression of IGF-1R correlated with unfavorable prognosis in BLCA, LIHC, and LUAD. **(D**, **E)** Kaplan-Meier method showed high expression of IGF-1R correlated with favorable prognosis in LAML and KIRC. **(F)** Forest plot displaying the impact of high expression of IGF-1R on OS across 33 cancer types using Cox regression model.

**Figures 2** and **3** summarized the results of OS analyses of IGF-1 and IGF-1R expression across the 33 cancer types. In univariate analysis, high expression of IGF-1 in tumor samples correlates with unfavorable prognosis in BLCA (HR = 1.09, *p* = 0.0012), CHOL (HR = 1.27, *p* = 0.0011) and LAML (HR = 3.88, *p* = 0.018); whereas, high expression of IGF-1 correlates with favorable prognosis in SARC (HR = 0.93, *p* = 0.00063) (**Figures 2A–D**). Cox regression model confirmed the prognostic impact of IGF-1 in BLCA (*p* = 4.4e−0.6), CHOL (*p* = 3.8e−0.2), LAML (*p* = 7.8e−0.3), and SARC (*p* = 4.2e−0.2) with the same trend (**Figure 2E**). On the other hand, high expression of IGF-1R in tumor samples correlates with unfavorable prognosis in BLCA (HR = 1.01, *p* = 0.045), LIHC (HR = 1.06, *p* = 0.013), and LUAD (HR = 1.01, *p* = 0.024); whereas, high expression of IGF-1R correlates with favorable prognosis in KIRC (HR = 0.97, *p* < 0.0001) and LAML (HR = 0.98, *p* = 0.0011) (**Figures 3A–E**). Cox regression model confirmed the prognostic impact of IGF-1R in BLCA (*p* = 4.0e−0.2), LIHC (*p* = 1.3e−0.2), LUAD (*p* = 2.7e−0.2), KIRC (*p* = 9.3e−0.8), and LAML (*p* = 3.2e−0.2) with the same trend (**Figure 3F**).

### Correlation of IGF-1/IGF-1R Expression Level and Immune Infiltrates

**Figure 4:**
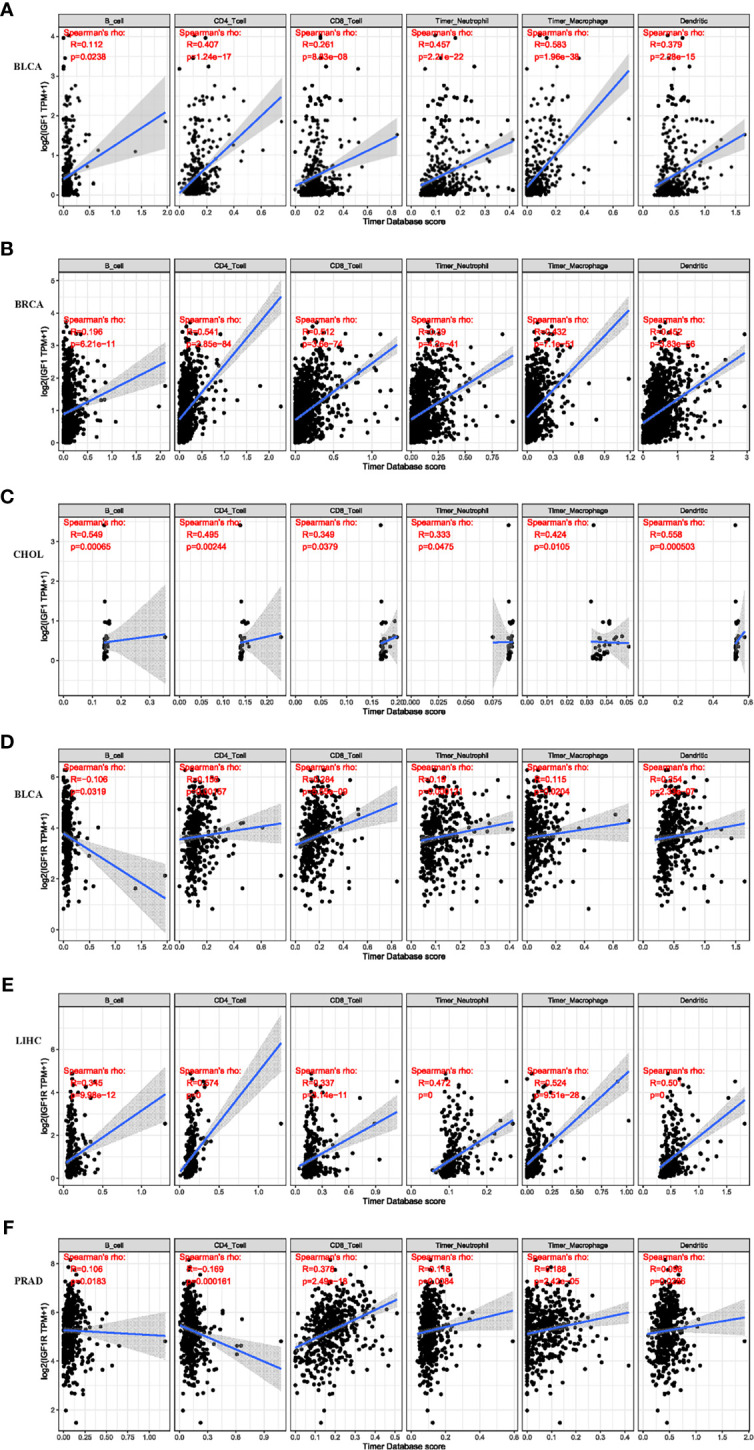
Correlation of IGF-1 expression level with immune infiltration level in BLCA, BRCA, and CHOL. Correlation of IGF-1R expression level with immune infiltration level in BLCA, LIHC, and PRAD. **(A–C)** IGF-1 expression is significantly positively correlated with CD4+ T cell, dendritic cell, and macrophage infiltration in BLCA, BRCA, and CHOL. **(D–F)** IGF-1R expression is significantly positively correlated with CD4+ T cell, dendritic cell, and macrophage infiltration in BLCA, LIHC, and PRAD.

Systemic analysis of immune infiltrates in different cancer types could be conducted by using a deconvolution statistical approach to infer tumor-infiltrating lymphocyte (TIL) counts based on gene expression data thanks to TIMER (https://cistrome.shinyapps.io/timer/). In this study, we analyzed the impact of IGF-1 and IGF-1R on the abundance of six immune infiltrates in cancers that harbor prognostic value, which are B cells, CD4+ cells, CD8+ cells, dendritic cells, macrophages, and neutrophils. The correlation between the expression of IGF-1/IGF-1R and the immune infiltration levels across diverse cancer types is derived from TIMER. As for IGF-1, three of the most significant associations were BLCA (**Figure 4A**), BRCA (**Figure 4B**), and CHOL (**Figure 4C**). While for IGF-1R, three of the most significant associations were BLCA (**Figure 4D**), LIHC (**Figure 4E**), and PRAD (**Figure 4F**). TIMER showed that both IGF-1 and IGF-1R are positively correlated with the abundance of CD4+ T cell, dendritic cells, and macrophages.

**Figure 5:**
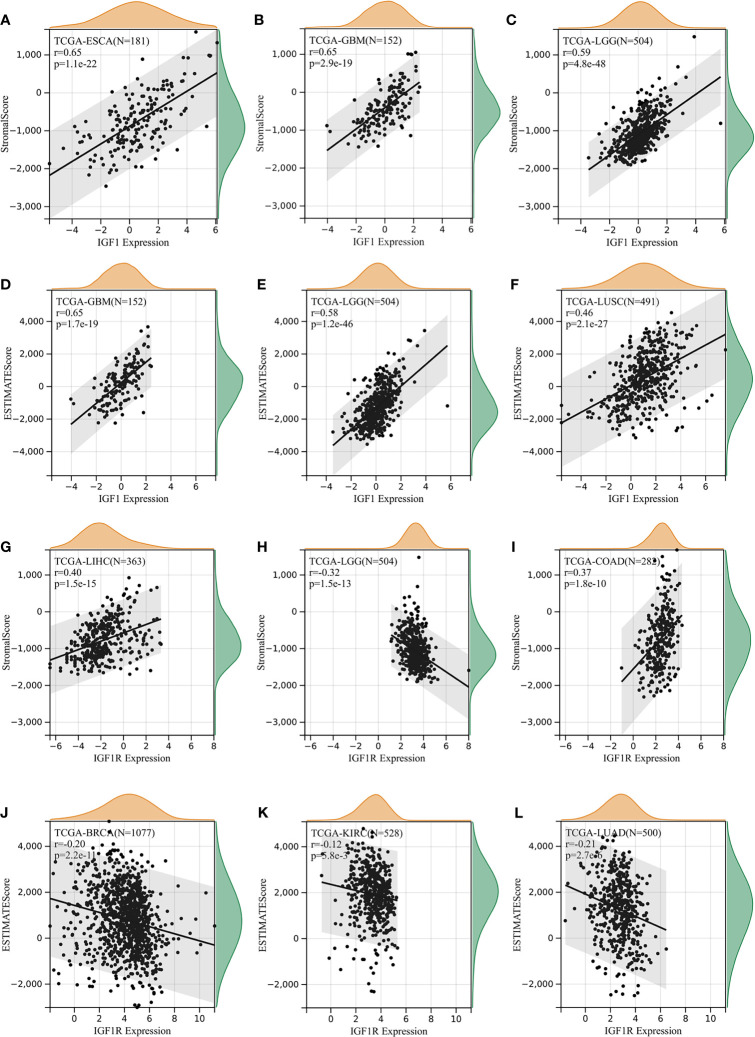
Correlation of IGF-1/IGF-1R expression with estimate score. **(A–C)** IGF-1 expression significantly correlated with stromal scores in esophageal carcinoma (ESCA), glioblastoma multiforme (GBM), and lower-grade glioma (LGG). **(D–F)** IGF-1 expression significantly correlated with immune scores in glioblastoma multiforme (GBM), lower-grade glioma (LGG), and lung squamous cell carcinoma (LUSC). **(G–I)** IGF-1R expression significantly correlated with stromal scores in liver hepatocellular carcinoma (LIHC), lower-grade glioma (LGG), and lung squamous cell carcinoma (LUSC). **(J–L)** IGF-1R expression significantly correlated with immune scores in breast invasive carcinoma (BRCA), kidney renal clear cell carcinoma (KIRC), and lung adenocarcinoma (LUAD).

The ESTIMATE method is developed to calculate the immune and stromal scores of cancer tissues. By adopting the ESTIMATE method, we calculated the immune, stromal scores, respectively. As for IGF-1, three of the most significant correlation according to stromal scores were found in ESCA, GBM, and LGG (**Figures 5A–C**), while three of the most significant correlation according to immune scores were found in GBM, LGG, and LUSC (**Figures 5D–F**). As for IGF-1R, three of the most significant correlation according to stromal scores were found in LIHC, LGG, and COAD (**Figures 5G–I**), while three of the most significant correlation according to immune scores were found in BRCA, KIRC, and LUAD (**Figures 5J–L**).

### Correlation Analysis on Checkpoint Gene Markers

**Figure 6:**
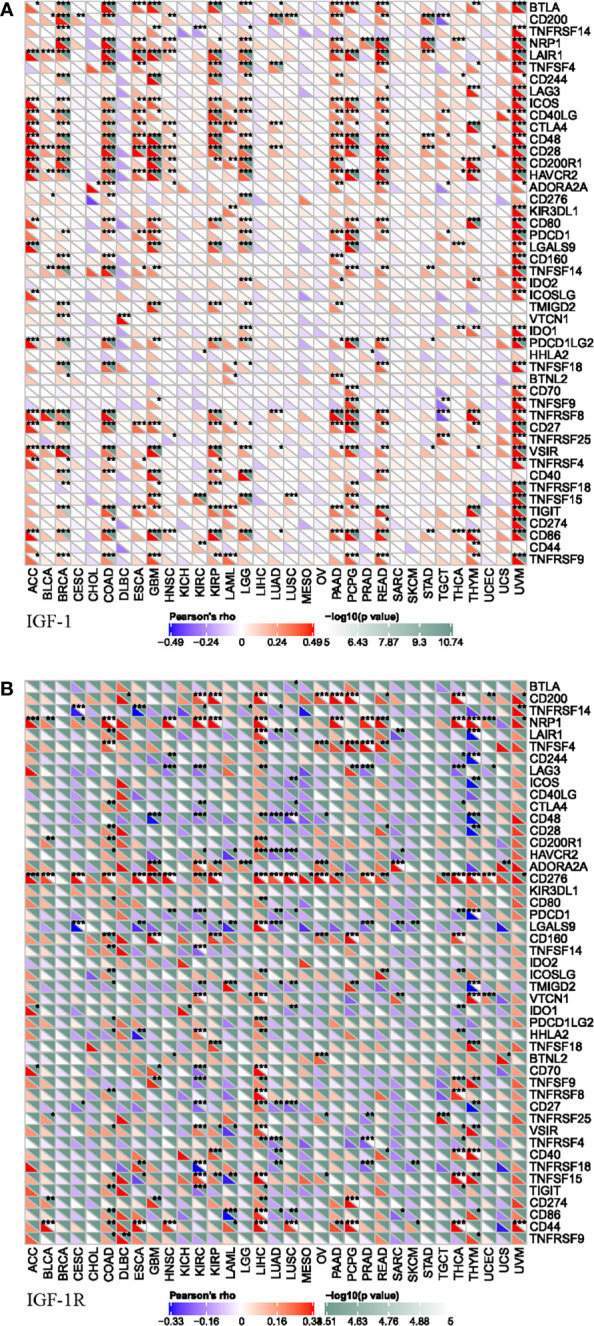
Correlation of IGF-1 **(A)** and IGF-1R **(B)** expressions with expression of immune checkpoint genes across 33 cancer types. “*, **, ***” means significant correlation p < 0.05, p < 0.01 and p < 0.001, respectively.

To further explored the potential mechanism of immune inhibition of IGF-1/IGF-1R signaling, the associations of IGF-1/IGF-1R expressions with multiple checkpoint markers were compared across different cancer types (**Figure 6**). Generally, IGF-1 expression positively correlates with the expression of LAIR1, ICOS, CD40LG, CTLA4, CD48, CD28, CD200R1, HAVCR2, and CD86 in the majority of 33 cancer types (**Figure 6A**). On the other hand, IGF-1R expression positively correlates with the expression of NRP1 and CD276 in the majority of 33 cancer types (**Figure 6B**).

### Correlation Analysis on TMB, MSI, MMR, and DNMT

**Figure 7:**
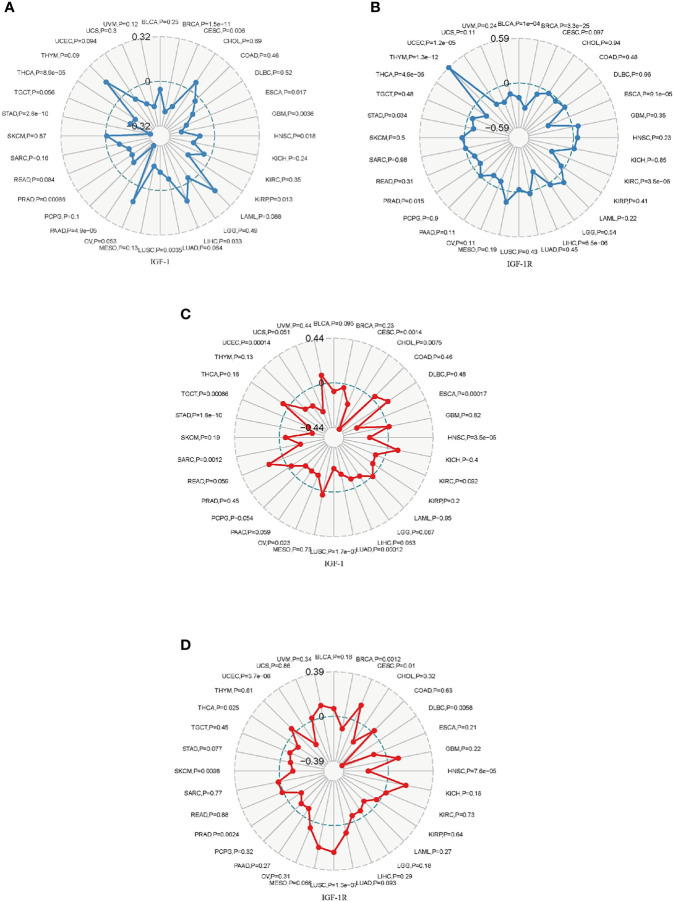
Radar map plotting the correlation of tumor mutation burden (TMB) **(A)** and microsatellite instability (MSI) **(B)** with IGF-1 expression across 33 cancer types. Radar map plotting the correlation of tumor mutation burden (TMB) **(C)** and microsatellite instability (MSI) **(D)** with IGF-1R expression across 33 cancer types.

Tumor mutational burden (TMB) is a quantifiable biomarker which is used to reflect the number of mutations contained in malignancies. Microsatellite instability (MSI) refers to the occurrence of new microsatellite alleles due to the insertion or deletion of duplicate units and cause changes in the length of a microsatellite compared with normal tissue. The association of TMB/MSI with IGF-1/IGF-1R expression was evaluated. Expression of IGF-1 positively correlated with TMB in THYM, CHOL, LAML, LIHC, and OV while negatively correlated with the TMB in BLCA, BRCA, CESC, COAD, DLBC, ESCA, GBM, HNSC, KICH, KIRC, KIRP, LGG, LUAD, LUSC, MESO, PAAD, PCPG, PRAD, READ, SARC, STAD, TGCT, THCA, UCEC, UCS, and UVMP (**Figure 7A**). Expression of IGF-1R positively correlated with TMB in GBM, HNSC, KICH, LAML, LGG, MESO, SKCM, and THYM while negatively correlated with TMB in BLCA, BRCA, CESC, COAD, ESCA, KIRC, KIRP, LIHC, OV, PAAD, PRAD, READ, STAD, TGCT, THCA, UCEC, UCS, and UVM (**Figure 7B**). The correlations of IGF-1/IGF-1R with MSI are shown in **Figures 7C, D**.

The correlation analysis between MMR genes (MLH1, MSH2, MSH6, PMS2, EPCAM) and IGF-1/IGF-1R expression was further performed. IGF-1 expression was correlated with at least one MMR genes in BRCA, COAD, HNSC, LIHC, PAAD, STAD, and TGCT (**Supplementary Figure S1**). IGF-1R expression was correlated with at least one MMR genes in almost all of the 33 cancer types except for BRCA, STAD, and UCS (**Supplementary Figure S2**). Moreover, the correlation analysis between DNMT (DNMT1, DNMT2, DNMT3A, and DNMT3B) and IGF-1/IGF-1R expression was also conducted. The result is as shown in **Supplementary Figures S3** and **S4**.

### Functional Analysis by Gene Set Enrichment Analysis

**Figure 8:**
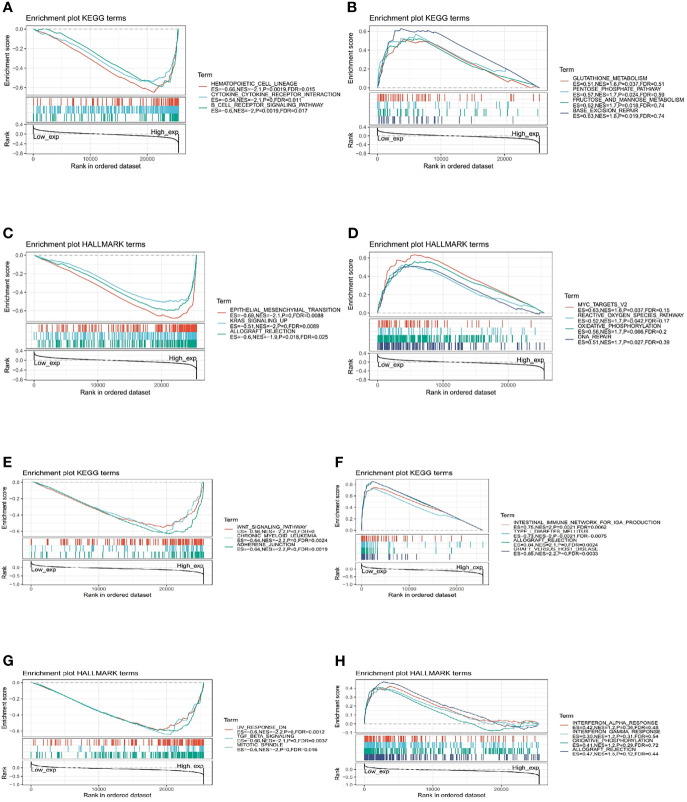
Functional Enrichment of KEGG and HALLMARK terms on IGF-1 and IGF-1R through GSEA. The top 3 negatively and positively enriched KEGG terms on IGF-1 are displayed in **(A, B)**, respectively. The top 3 negatively and positively enriched KEGG terms on IGF-1 are displayed in **(C, D)**, respectively. The top 3 negatively and positively enriched KEGG terms on IGF-1R are displayed in **(E, F)**, respectively. The top 3 negatively and positively enriched KEGG terms on IGF-1R are displayed in **(G, H)**, respectively.

The biological role of IGF-1 and IGF-1R were illustrated through GSEA. The pan-cancer functional KEGG and HALLMARK terms of IGF-1/IGF-1R are shown in **Figure 8**, respectively. Generally, the top 3 negatively enriched KEGG terms in high IGF-1 subgroups were hematopoietic cell lineage, cytokine cytokine receptor interaction, and B-cell receptor signaling pathway (**Figure 8A**), and the top positively enriched KEGG terms were glutathione metabolism, pentose phosphate pathway, fructose and mannose metabolism, and base excision repair (**Figure 8B**). The top 3 negatively enriched HALLMARK terms in high IGF-1 subgroups were epithelial mesenchymal transition, KRAS upsignaling, and allograft rejection (**Figure 8C**), and the top positively enriched HALLMARK terms were MYC targets V2, reactive oxygen species pathway, oxidative phosphorylation, and DNA repair (**Figure 8D**). As for IGF-1R, the top 3 negatively enriched KEGG terms were WNT signaling pathway, chronic myeloid leukemia, and adherens junction (**Figure 8E**), and the top positively enriched KEGG terms were intestinal immune network for IgA production, type I diabetes mellitus, allograft rejection, and graft-versus-host disease (**Figure 8F**). The top 3 negatively enriched HALLMARK terms in high IGF-1R subgroups were UV response DN, TGF beta signaling, and mitotic spindle (**Figure 8G**), and the top positively enriched HALLMARK terms were interferon alpha response, interferon gamma response, oxidative phosphorylation, and allograft rejection (**Figure 8H**).

## Discussion

The present work illustrated a comprehensive workflow for pan-cancer analysis and thoroughly investigated the role of IGF-1 and IGF-1R in cancers. The prognostic impact of IGF-1 and IGF-1R expression among different cancer types was reported. It was found that most cancer types showed a higher IGF-1/IGF-1R alteration frequency and the expression of IGF-1 and IGF-1R served as the prognostic factor in some cancer types, including BLCA and LAML upon both Cox and KM survival analyses. However, the relationship between IGF-1/IGF-1R overexpression and tumor immunity was still unclear. Secondly, though IGF-1 and IGF-1R share the same signaling pathway, whether they have different prognostic and immunologic values in different types of cancer needs further investigation. Moreover, the relationship between IGF1/IGF1R expression and tumor prognoses, such as TMB, MSI, MMR, and DNMT, which can predict the efficacy of immunotherapy, remains unclear. Base on this, our bioinformatics analysis studied the IGF-1/IGF-1R expression-associated KEGG terms and HALLMARK pathways.

Our study showed that IGF-1 and IGF-1R harbor distinct prognostic values among different cancer types. The low expression of IGF-1 served as a favorable prognostic factor in some cancer types, including BLCA, CHOL, LAML, and UVM; whereas in SARC, high expression of IGF-1 served as a favorable prognostic factor. On the other hand, high expression of IGF-1R served as a significant prognostic factor in such cancer types including KIRC and LAML; whereas in BLCA, LIHC, and LUAD, low expression of IGF-1R served as a favorable prognostic factor. Previous study has proven that the epithelial-mesenchymal transition (EMT) process of gastric cancer cells could be induced by IGF-1 through the IGF-1R/STAT3 signaling pathway so that cancer cells would achieve metastasis (16). It has also been found that in liver cancer, IGF-1 promotes the invasion and metastasis of liver cancer cells by inhibiting the degradation of cathepsin B (17). Wu et al. proved that the increasing secretion of IGF-1 and CCL20 promotes brain metastasis of lung cancer cells by polarizing microglia and suppressing innate immune function (18). Studies done by Somri-Gannam et al. provide evidence that IGF-1R axis inhibition could be a therapeutic strategy for ovarian cancer by restoring dendritic cell (DC)-mediated antitumor immunity (19). Therefore, the distinct prognostic value of changeable IGF-1/IGF-1R expression across different cancers may result from its synthetic effect of immune suppressive activity and tumor suppressive activity in each cancer type. The distinct prognostic value of IGF-1 and IGF-1R expression may give rise to some new researches and questions for discussion. Both IGF-1 and IGF-1R are positively correlated with the abundance of CD4+ T cell, dendritic cells, and macrophages. Cellular infiltration of CD4+ T cell, dendritic cells, and macrophages would lead to immunosuppressive tumor microenvironment and cause unfavorable prognosis. The expression of IGF-1 is positively correlated with the expression of most immune checkpoint genes in UVM. The expression of IGF-1R is positively correlated with the expression of CD276 in most cancer types, and since CD276 is related to immunosuppression, this may explain why the increased expression of IGF-1R in tumor has an impact on the prognosis.

The expressions of IGF-1 and IGF-1R were correlated with TMB and MSI in some cancer types. TMB could impact the patient response to immune checkpoint inhibitors through affecting the generation possibility of immunogenic peptide (20). MSI is a vital index to predict tumor genesis and development (21). The NCCN guidelines have recommended MSI testing for all rectum adenocarcinoma (READ) subtypes, and the READ mortality can be reduced by the early detection of MSI (22). FDA has approved the use of Keytruda for the treatment of MSI-H solid tumors. As a result, TMB and MSI can serve as the predicting factors for efficacy. The expressions of IGF-1/IGF-1R share negative correlation with TMB and MSI in most cancer types, which means high expression of IGF-1/IGF-1R indicates immunosuppression. Our findings provide clues on the correlation between IGF-1/IGF-1R expression and cancer immunity and suggest that it could be a potential predictive maker of the efficacy of immunotherapy. Our study systematically compared the immune effects of IGF-1 and IGF1-R, which is conducive to localize the target molecules more accurately. A series of drugs targeting IGF-1/IGF1-R in the treatment of cancer are in clinical trials. The associated results may be released in the next 5 years.

Our study has several limitations. First, the result of our study should be interpreted with caution since checkpoint inhibitor treatment has not been analyzed in our study. Second, the result of our study lacks external validation in other public datasets. We need clinical specimens from our center for further verification. Only the gene expression level was analyzed in this study; we may conduct a more comprehensive analysis from the perspective of single and multiple omics. More efforts are needed to undermine the value of IGF-1/IGF-1R as a potential target of immunotherapy.

To conclude, our comprehensive pan-cancer analysis has characterized IGF-1/IGF-1R expression in different cancer cell lines and tissues. According to our results, IGF-1 and IGF-1R can serve as a valuable prognostic biomarker in some cancer types. They are also related to cancer immunity and could be potential predictive maker of the efficacy of immunotherapy, which may help develop the new targeted treatment.

## Data Availability Statement

The original contributions presented in the study are included in the article/**Supplementary Material**. Further inquiries can be directed to the corresponding authors.

## Author Contributions

LS and PZ designed and implemented the study design. YZ, CG, FC, YW, and SC participated in data analysis. XH, JM, ZQ, and WF managed and advised on the project. YZ, CG, FC, and YW wrote the paper. All authors contributed to the article and approved the submitted version.

## Funding

This study was supported by the National Outstanding Youth Science Fund Project of Natural Science Foundation of China (81801804).

## Conflict of Interest

The authors declare that the research was conducted in the absence of any commercial or financial relationships that could be construed as a potential conflict of interest.

## Publisher’s Note

All claims expressed in this article are solely those of the authors and do not necessarily represent those of their affiliated organizations, or those of the publisher, the editors and the reviewers. Any product that may be evaluated in this article, or claim that may be made by its manufacturer, is not guaranteed or endorsed by the publisher.

## Supplementary Material

Supplementary Figure 1 Correlations of IGF-1 expression with MMR genes.

Supplementary Figure 2 Correlations of IGF-1R expression with MMR genes.

Supplementary Figure 3 Correlations of IGF-1 expression with DNMT.

Supplementary Figure 4 Correlations of IGF-1R expression with DNMT.
