# Supplementary figures and images for: Pan-Cancer Analysis of IGF-1 and IGF-1R as Potential Prognostic Biomarkers and Immunotherapy Targets
Source: Front Oncol. 2021 Nov 5;11:755341. doi: 10.3389/fonc.2021.755341 (PMC8602838; doi:10.3389/fonc.2021.755341)

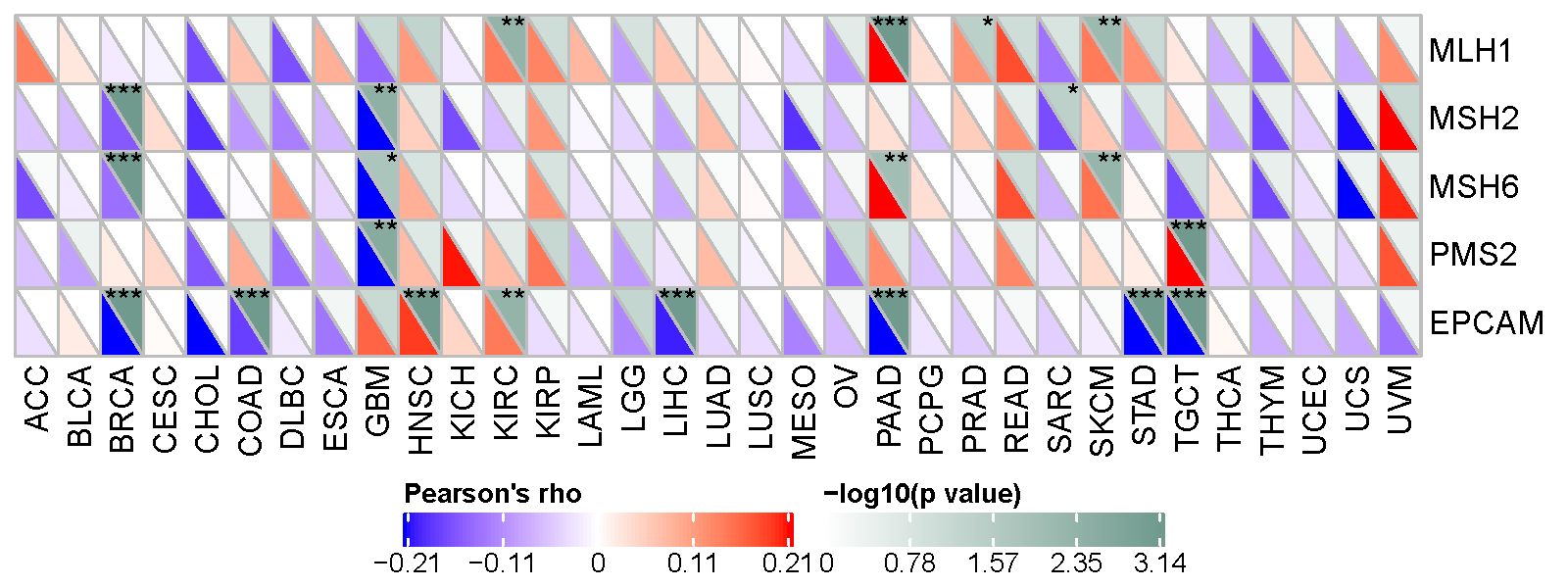

Supplement: Supplementary Figure 1 — Correlations of IGF-1 expression with MMR genes. [file Image_1.tif]

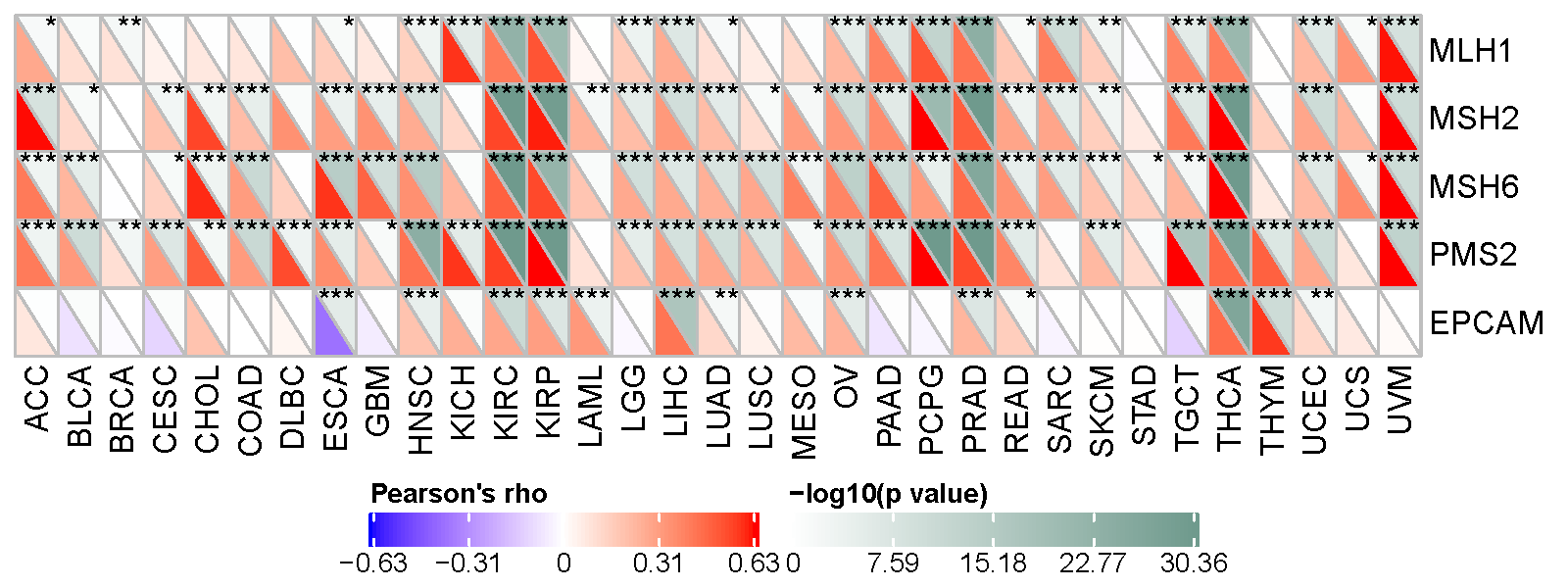

Supplement: Supplementary Figure 2 — Correlations of IGF-1R expression with MMR genes. [file Image_2.tif]

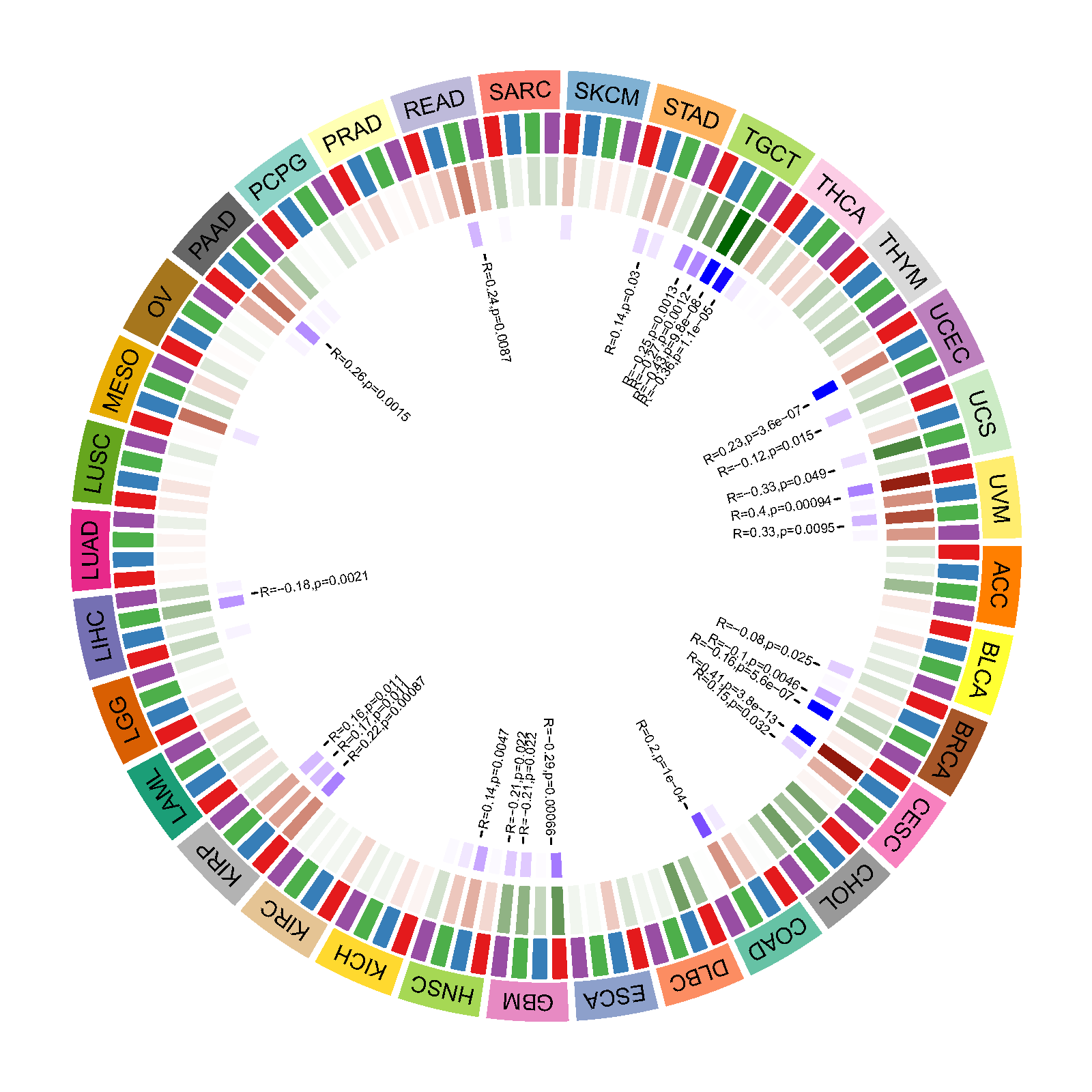

Supplement: Supplementary Figure 3 — Correlations of IGF-1 expression with DNMT. [file Image_3.tif]

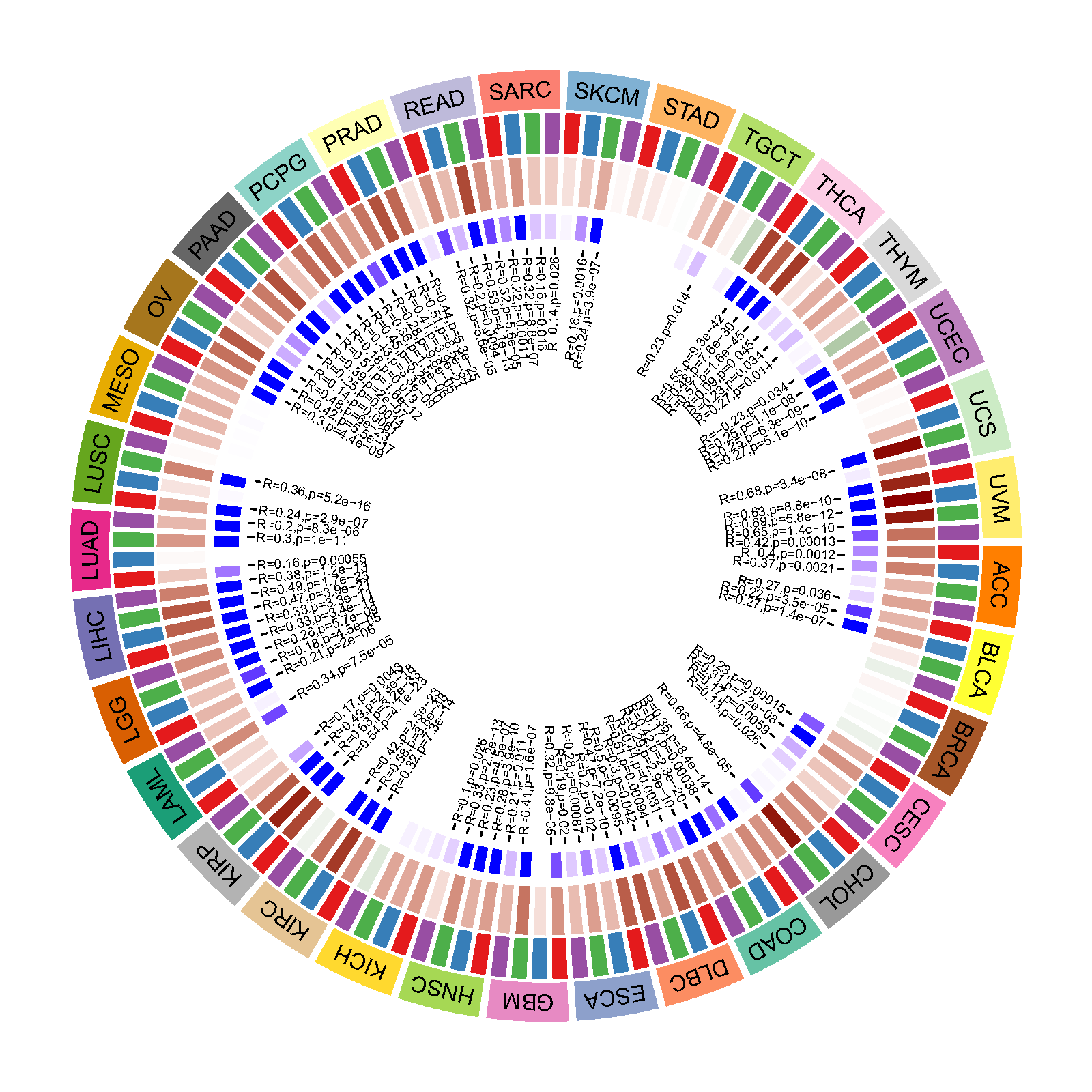

Supplement: Supplementary Figure 4 — Correlations of IGF-1R expression with DNMT. [file Image_4.tif]

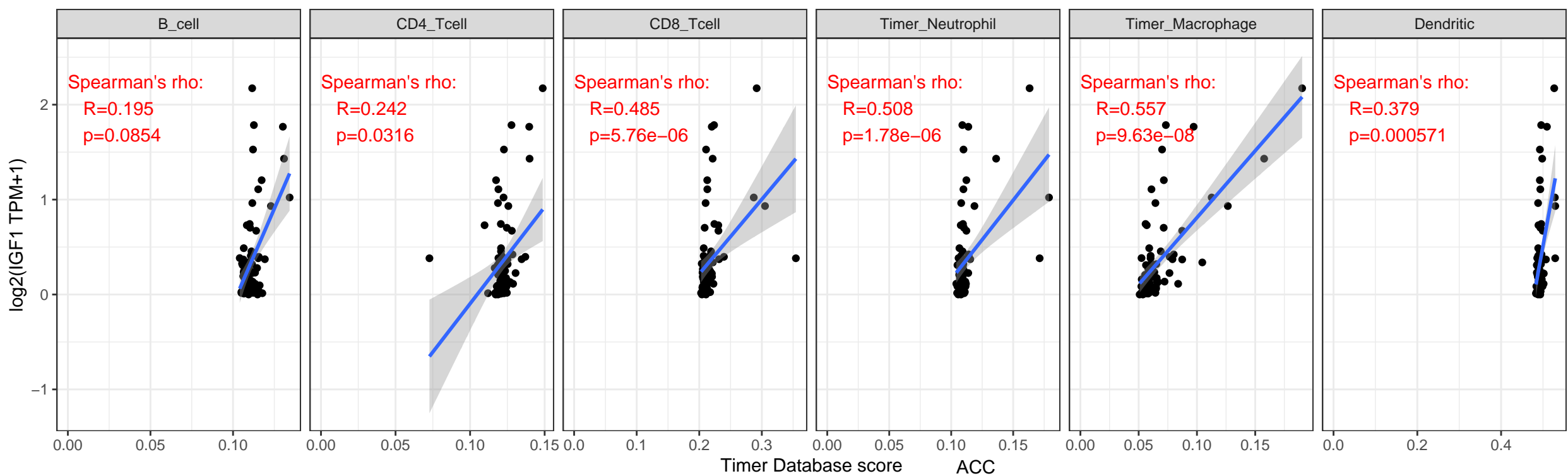

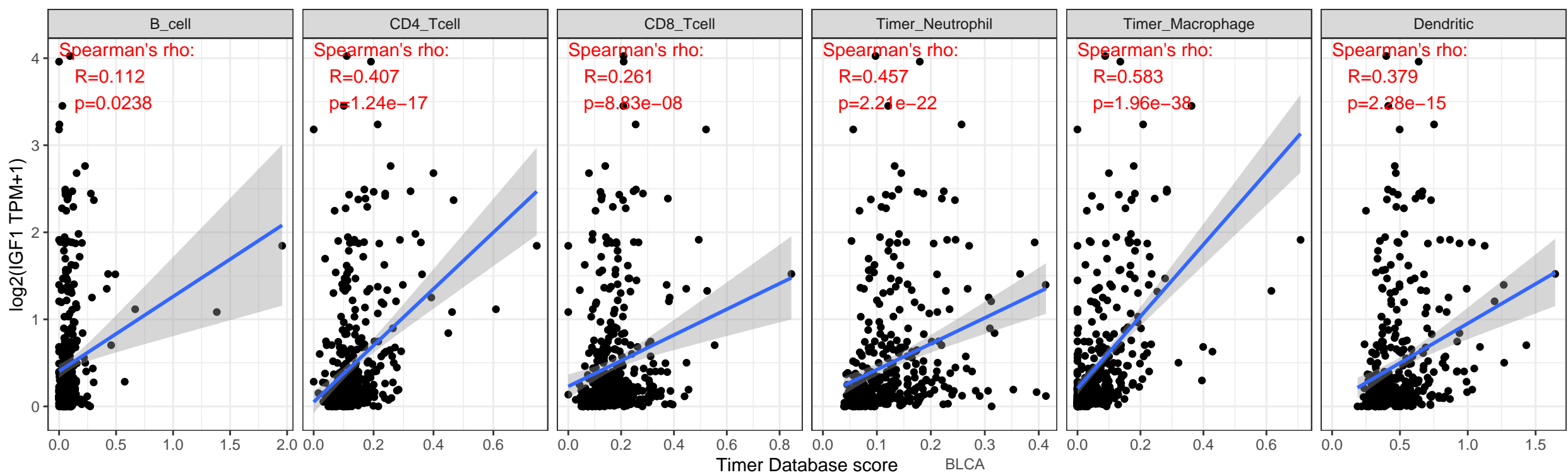

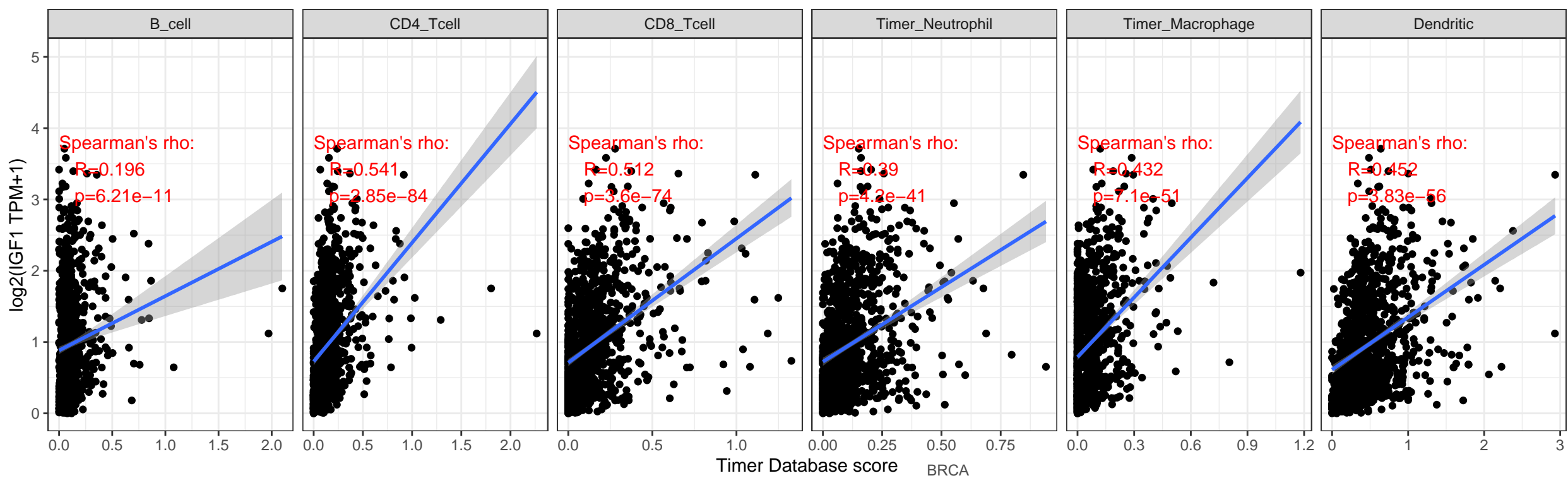

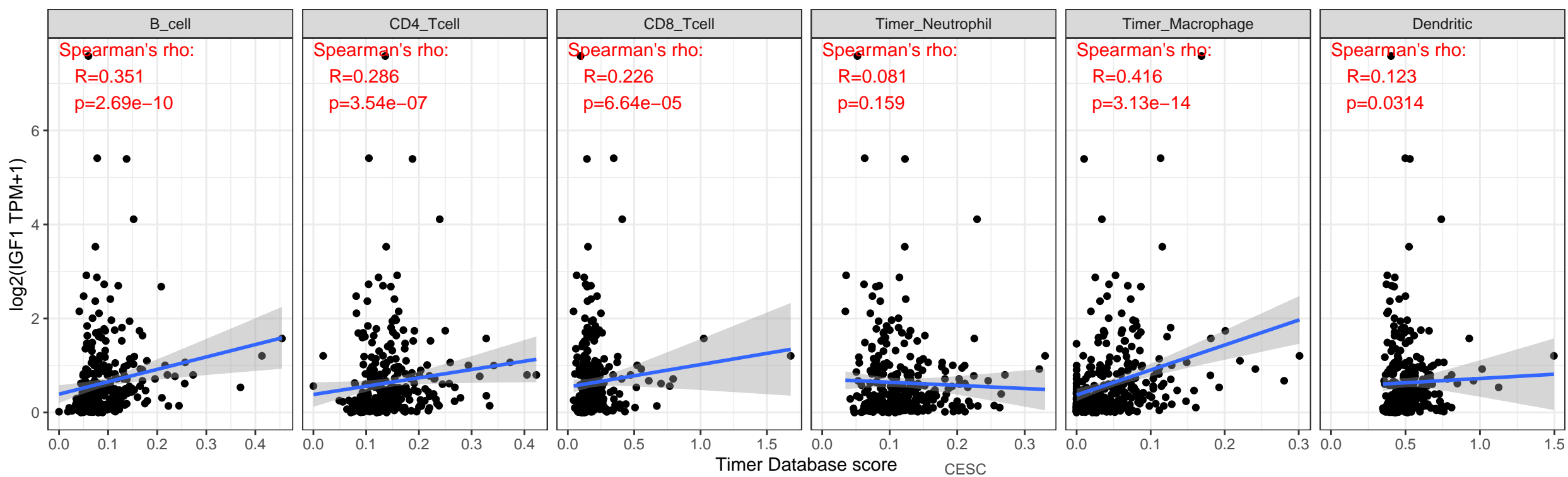

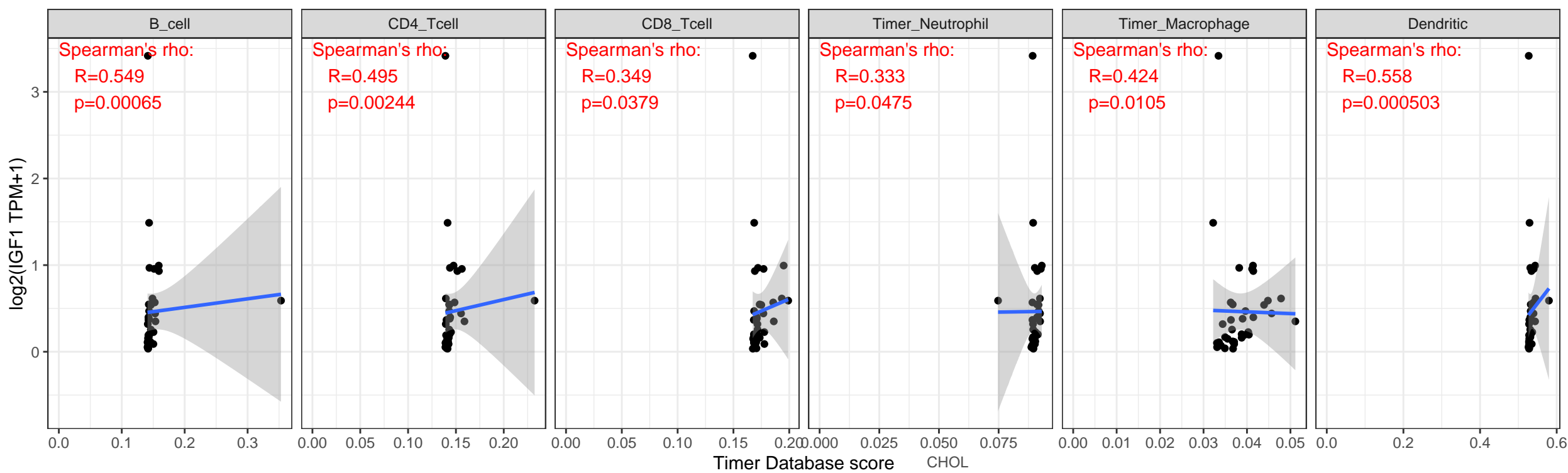

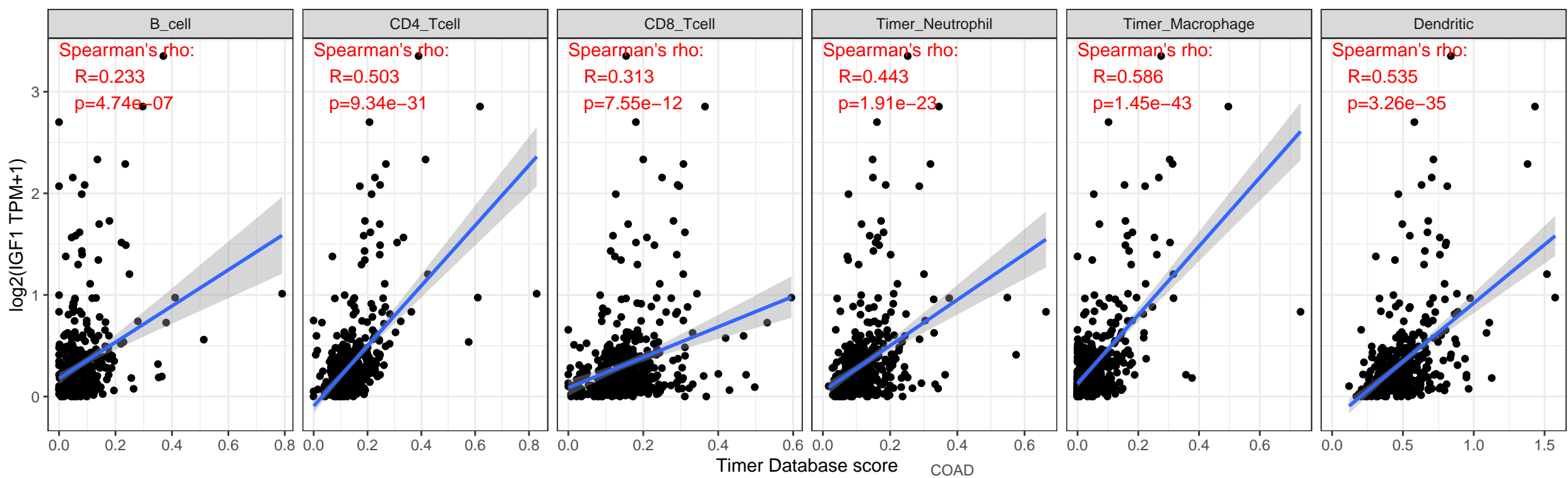

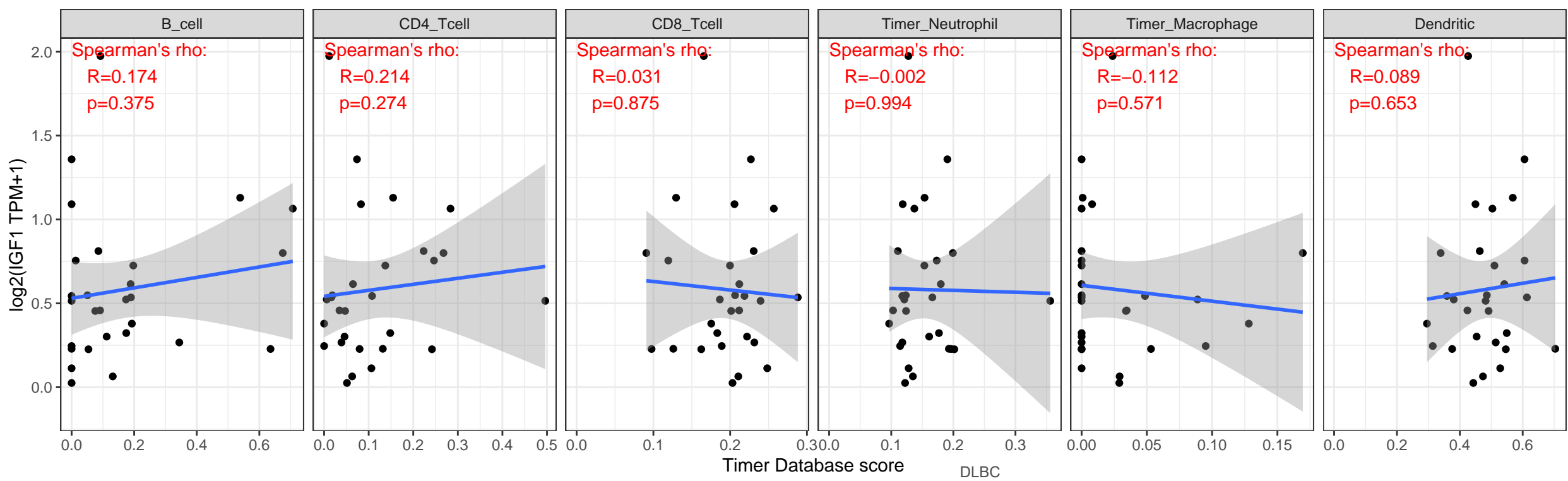

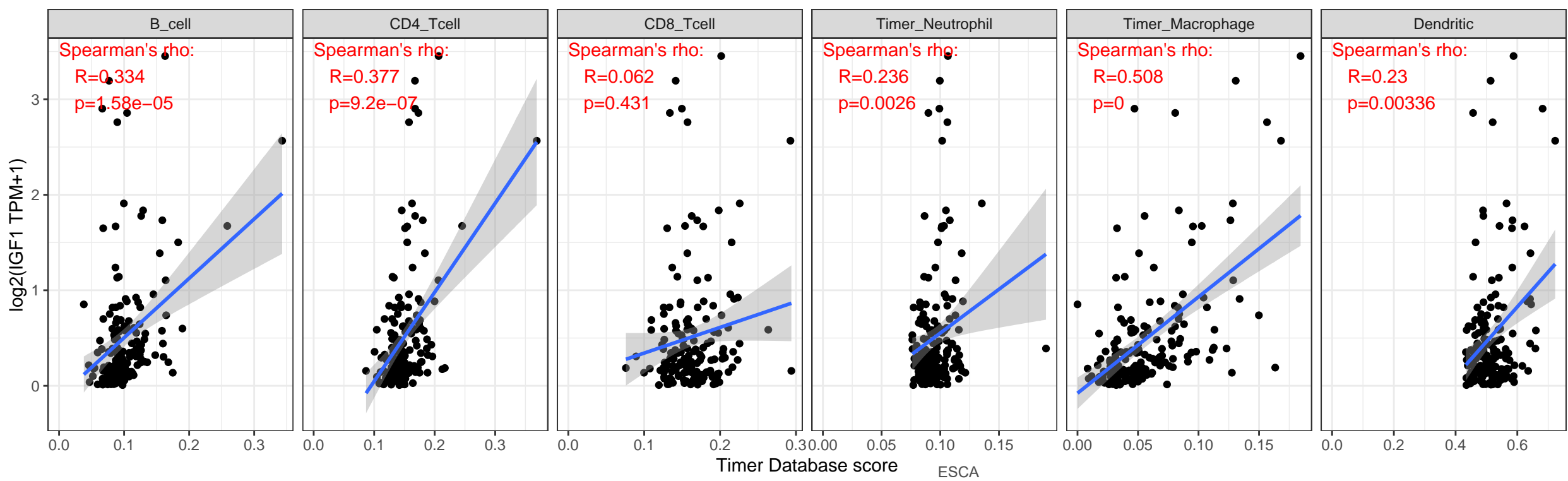

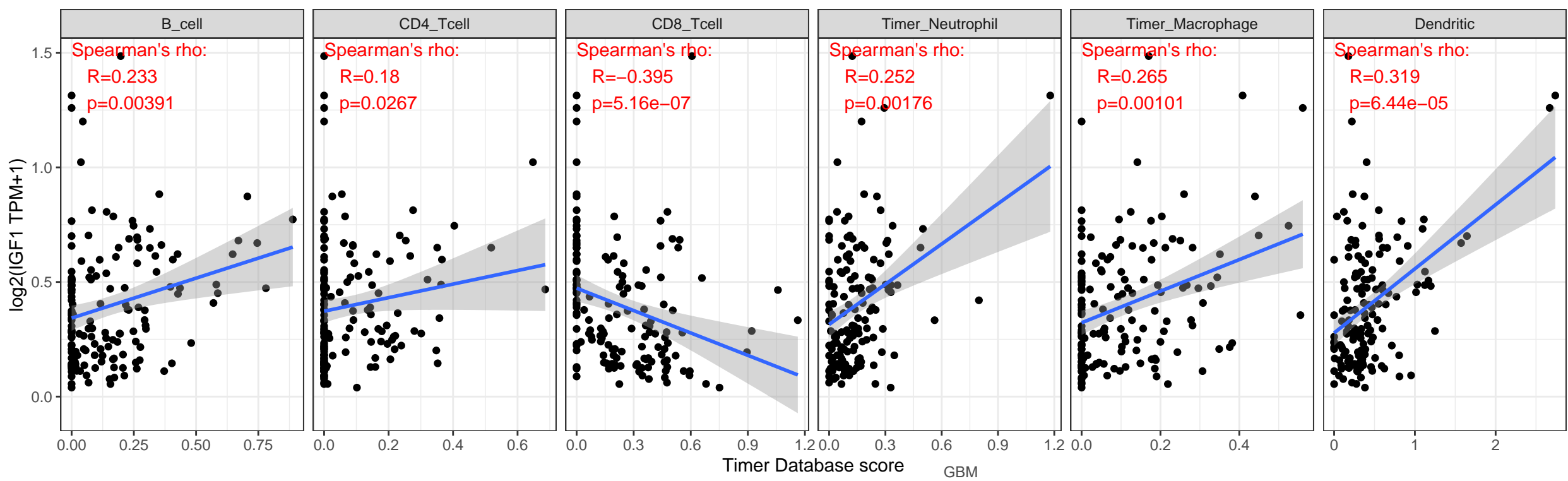

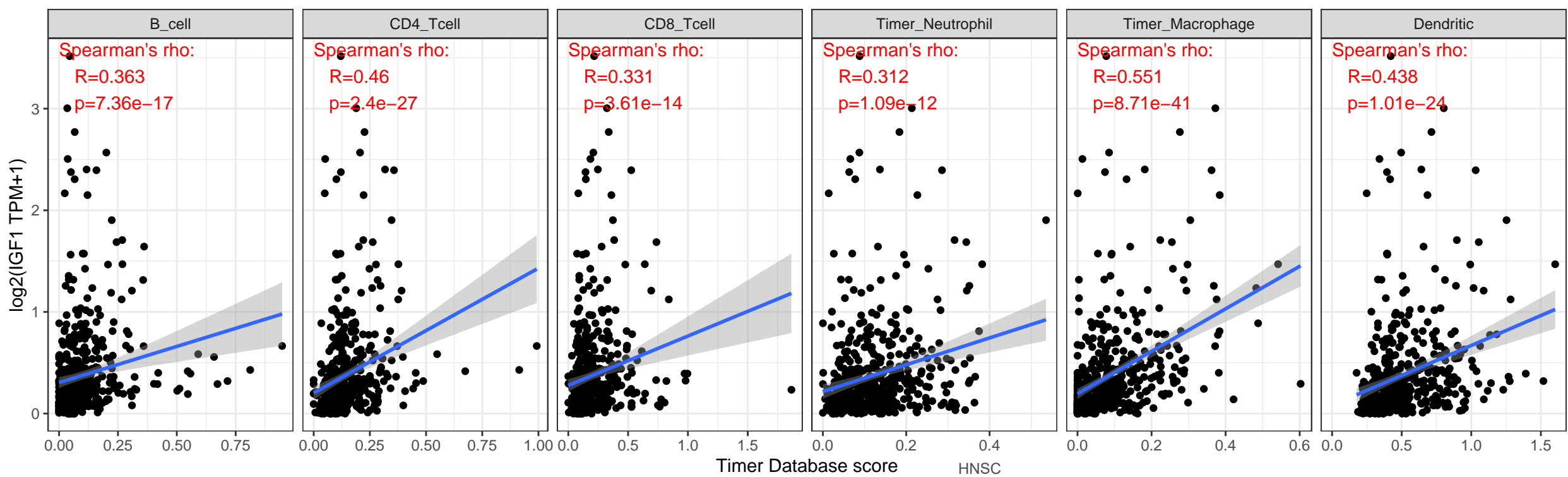

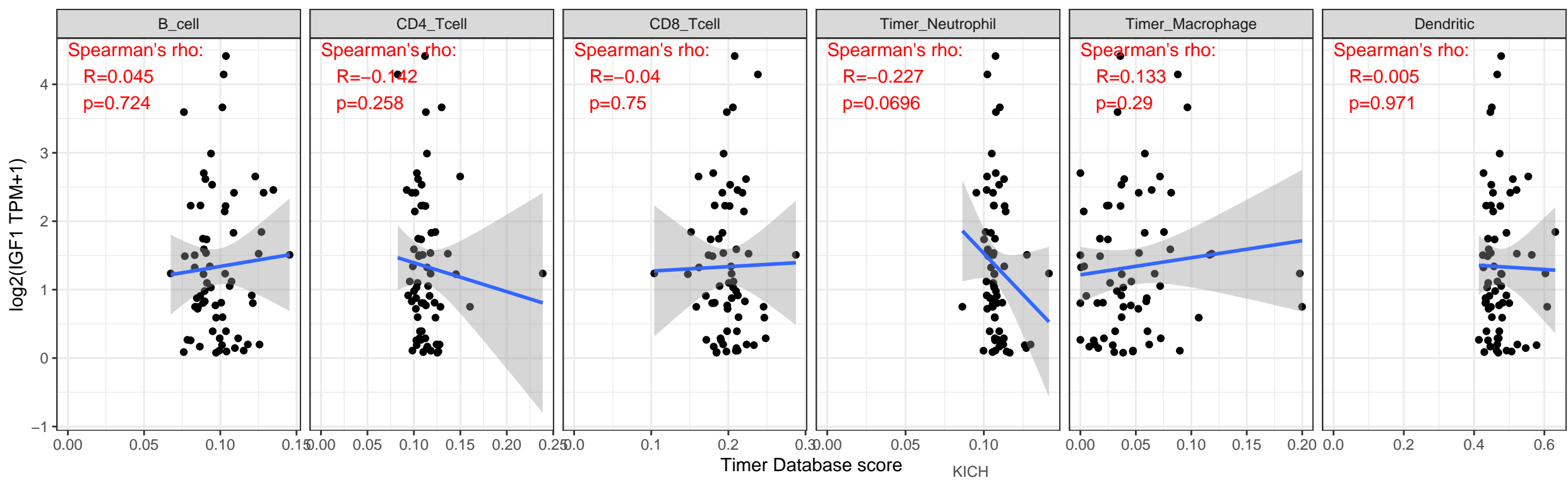

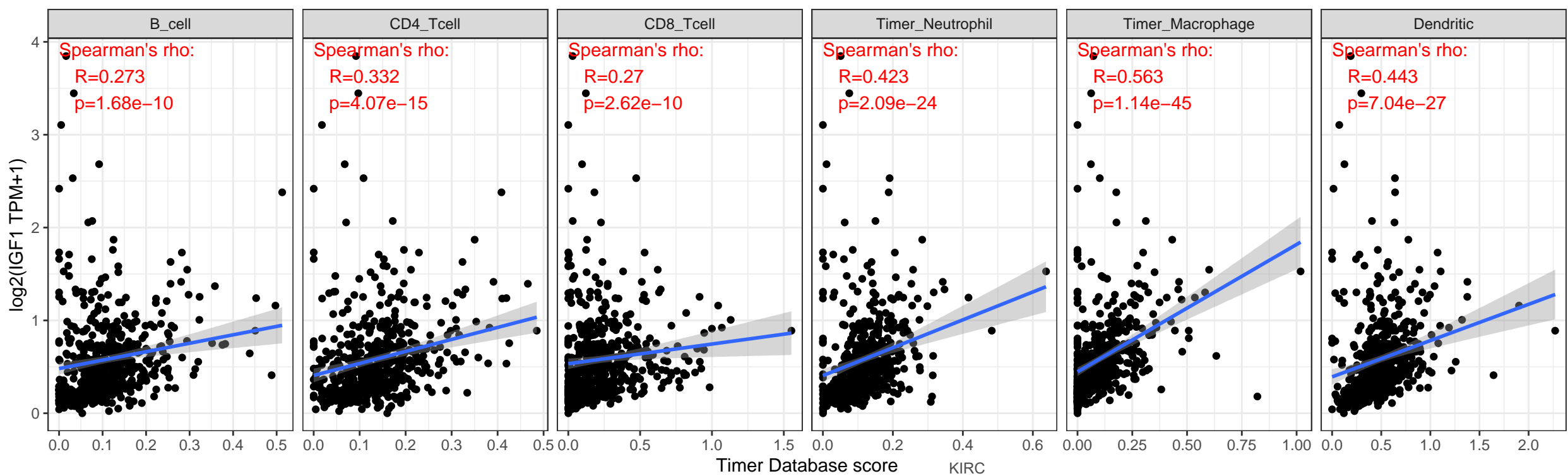

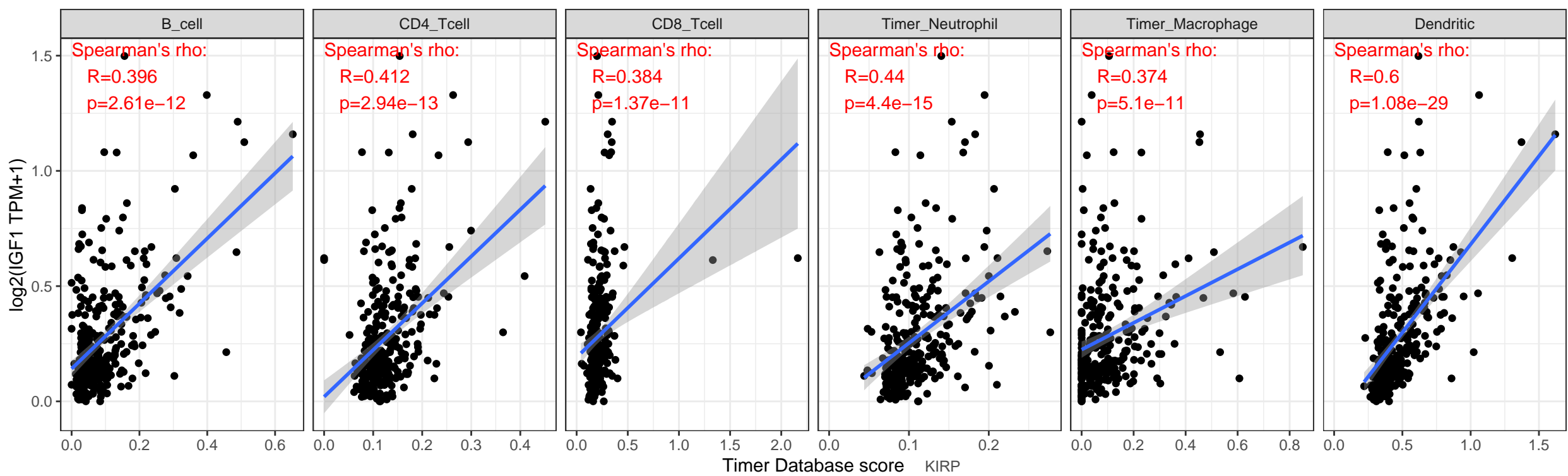

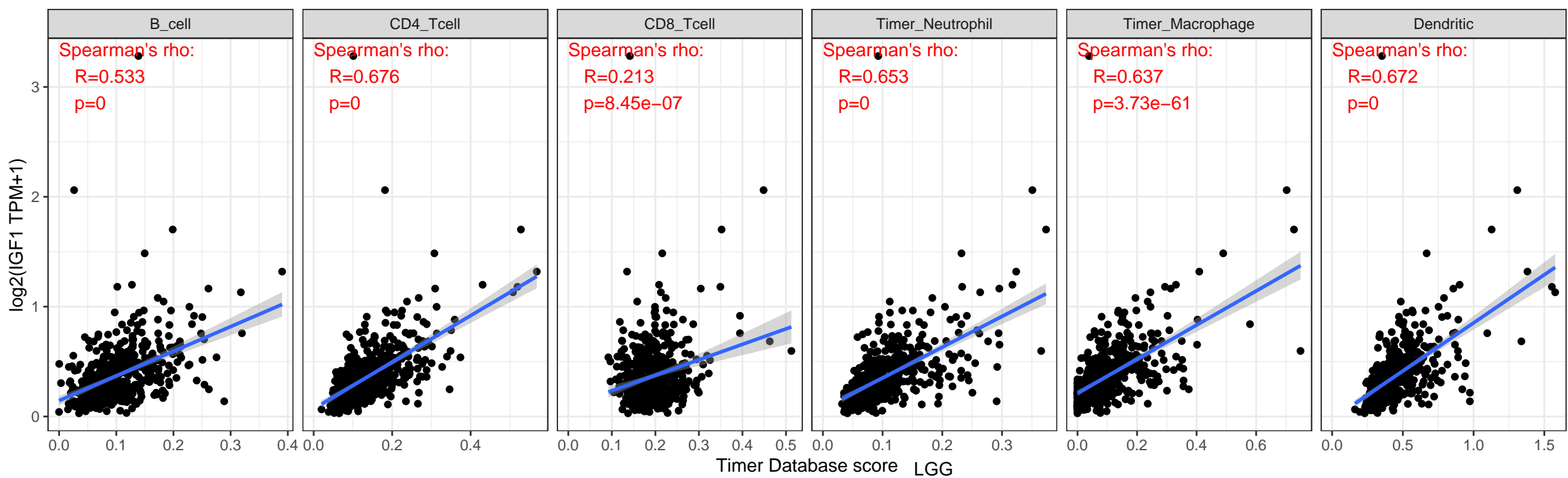

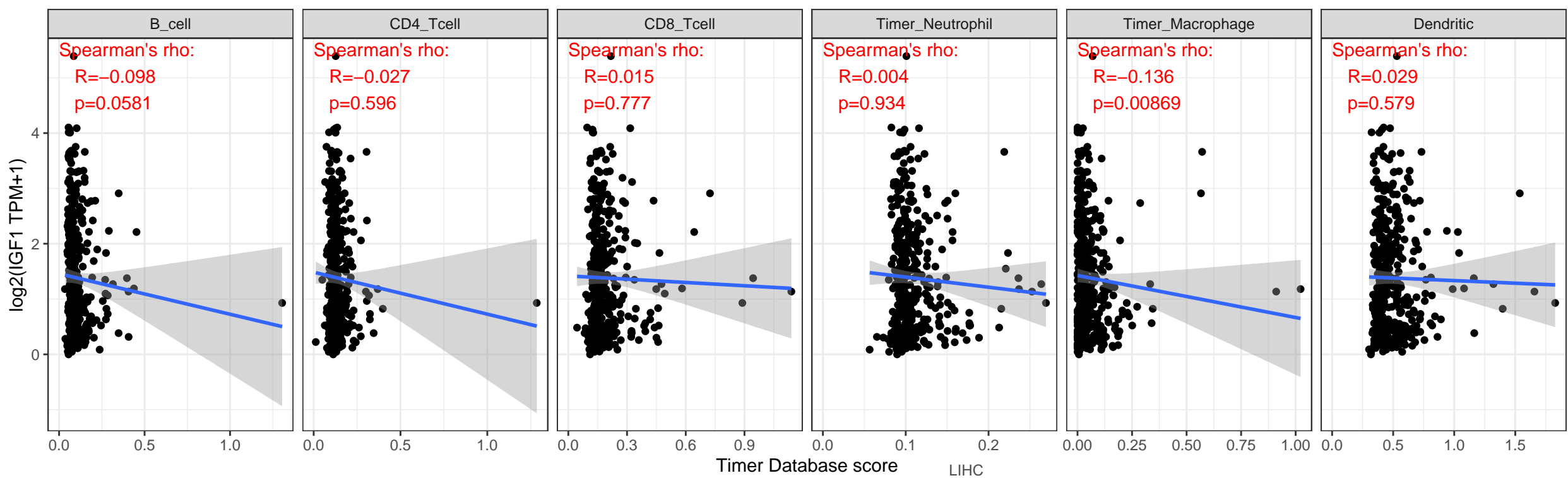

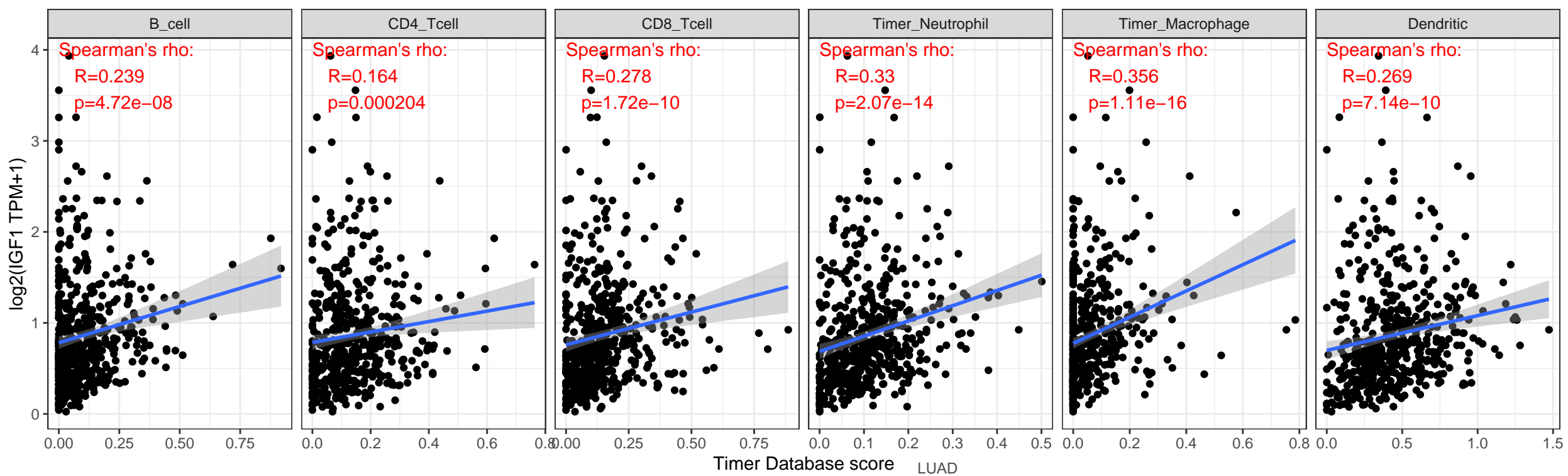

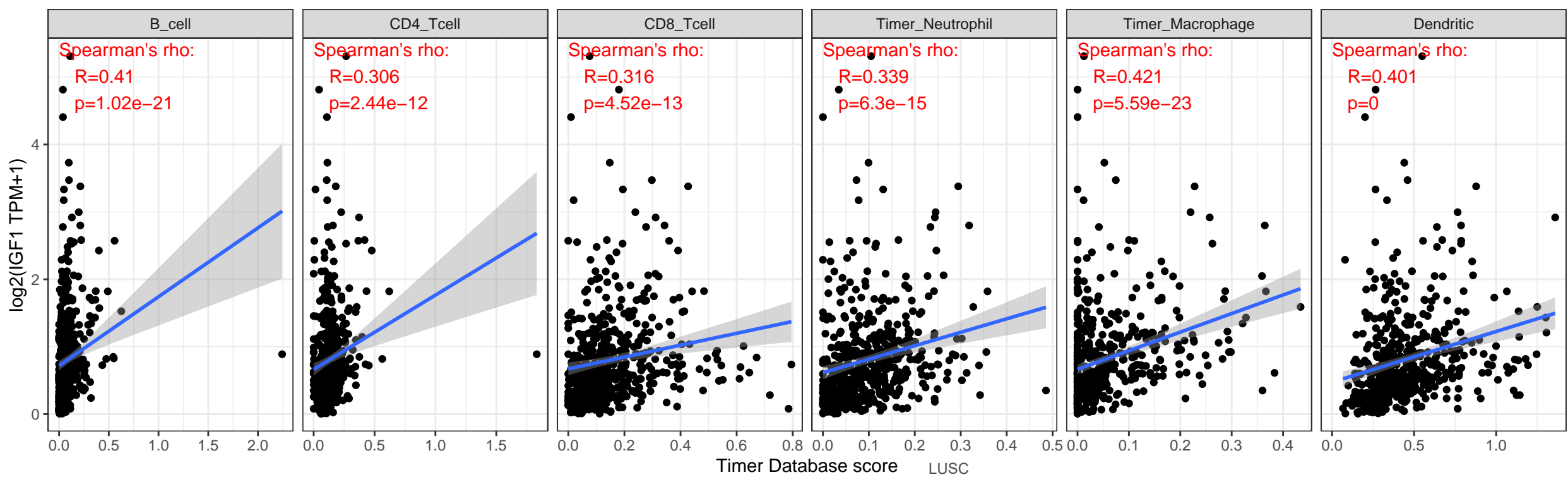

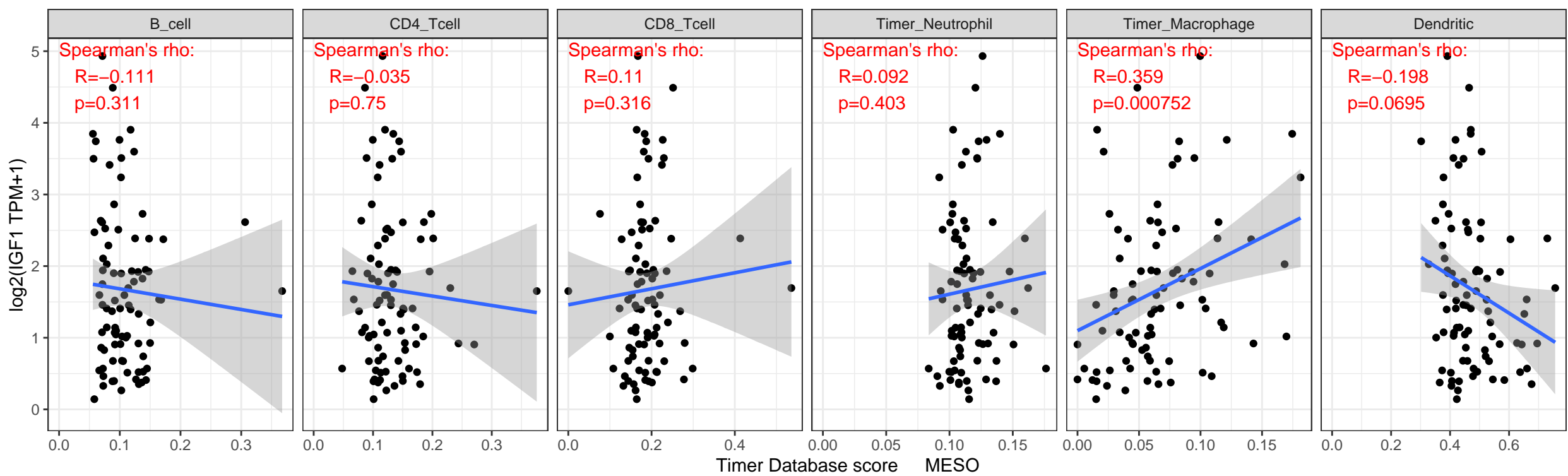

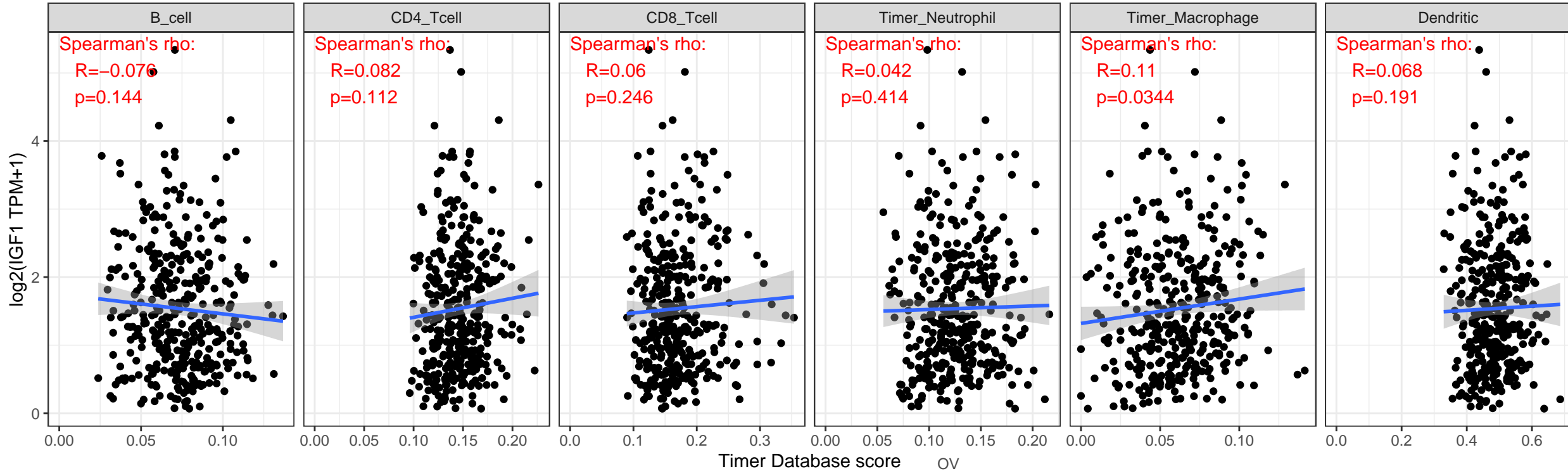

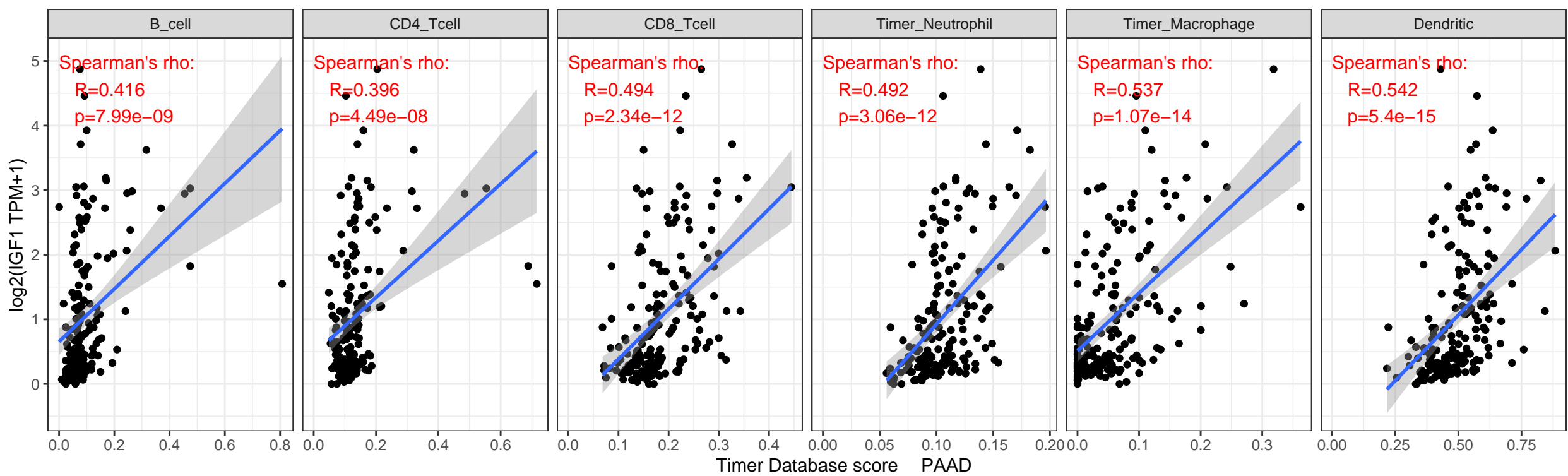

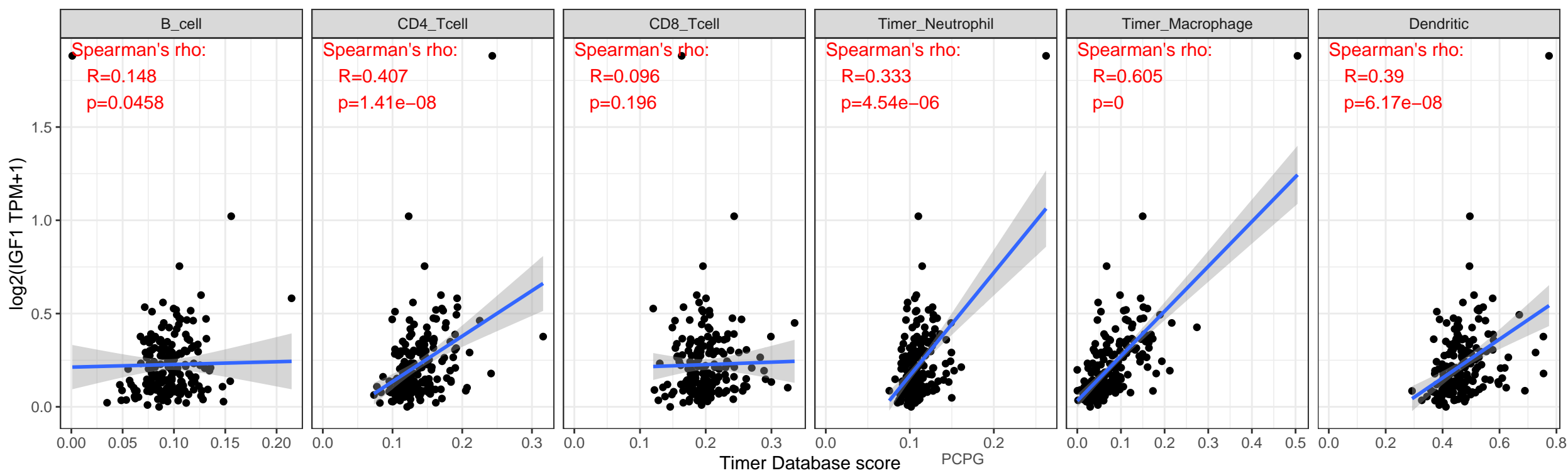

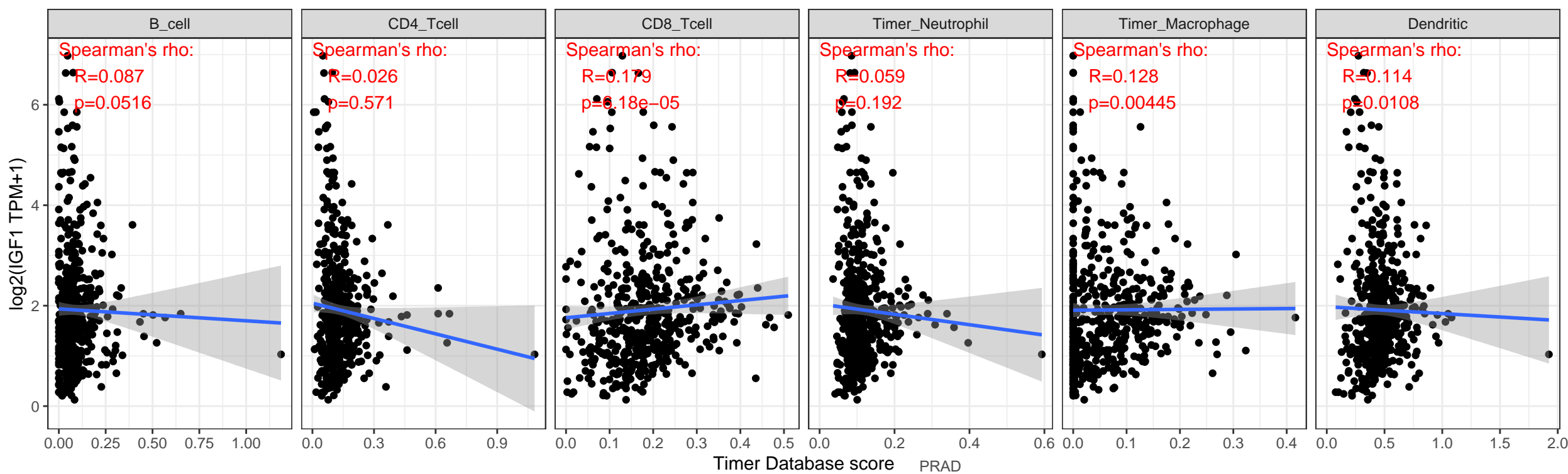

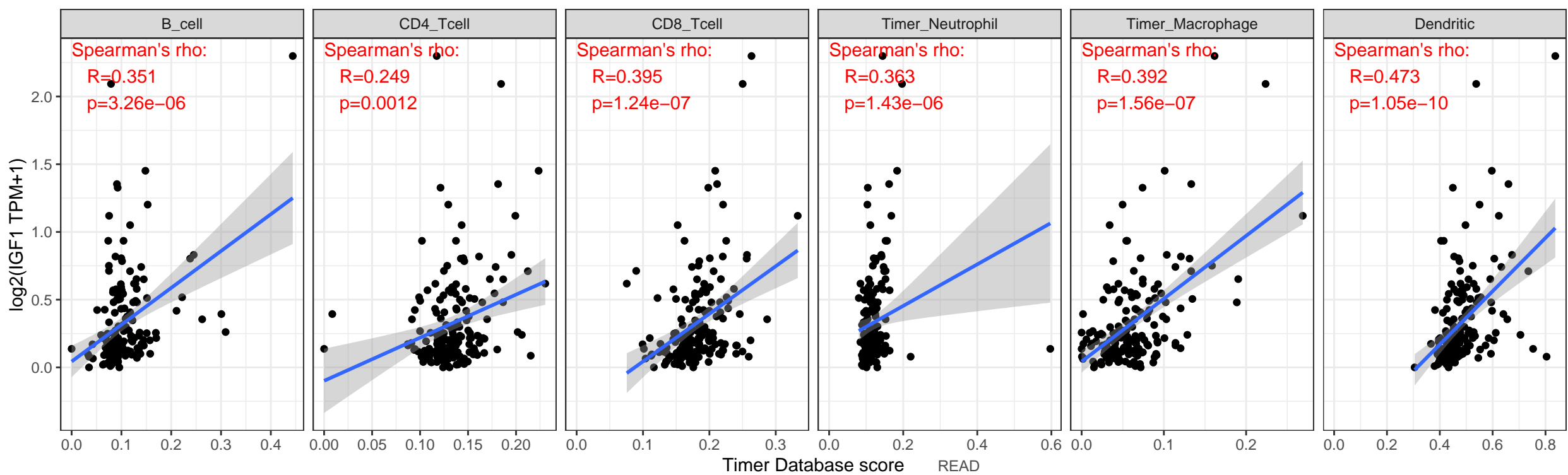

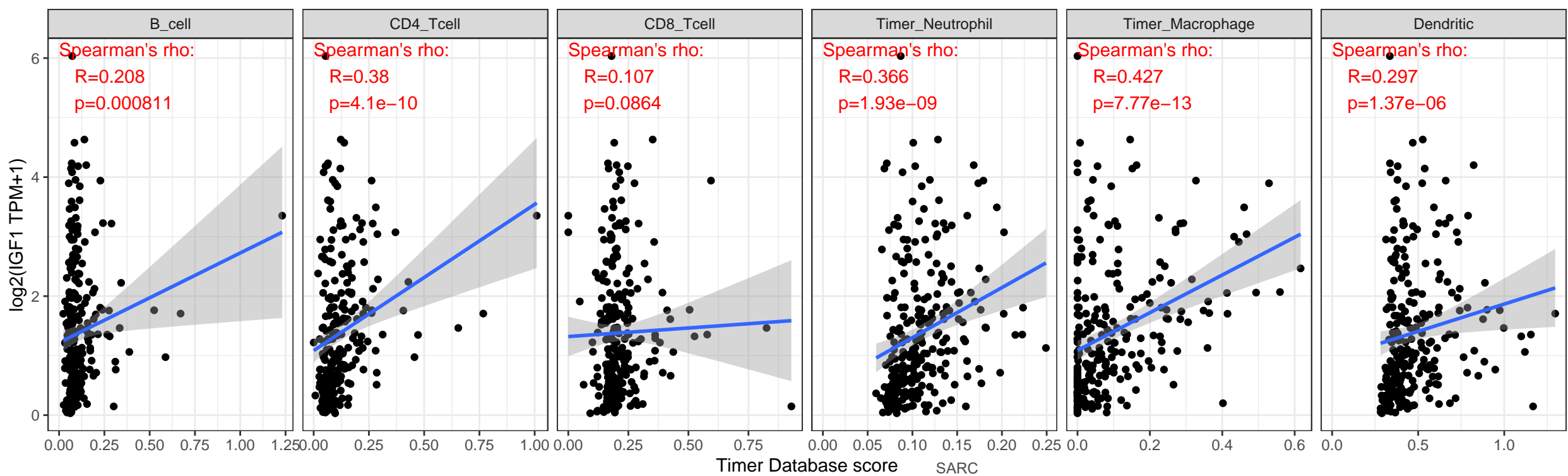

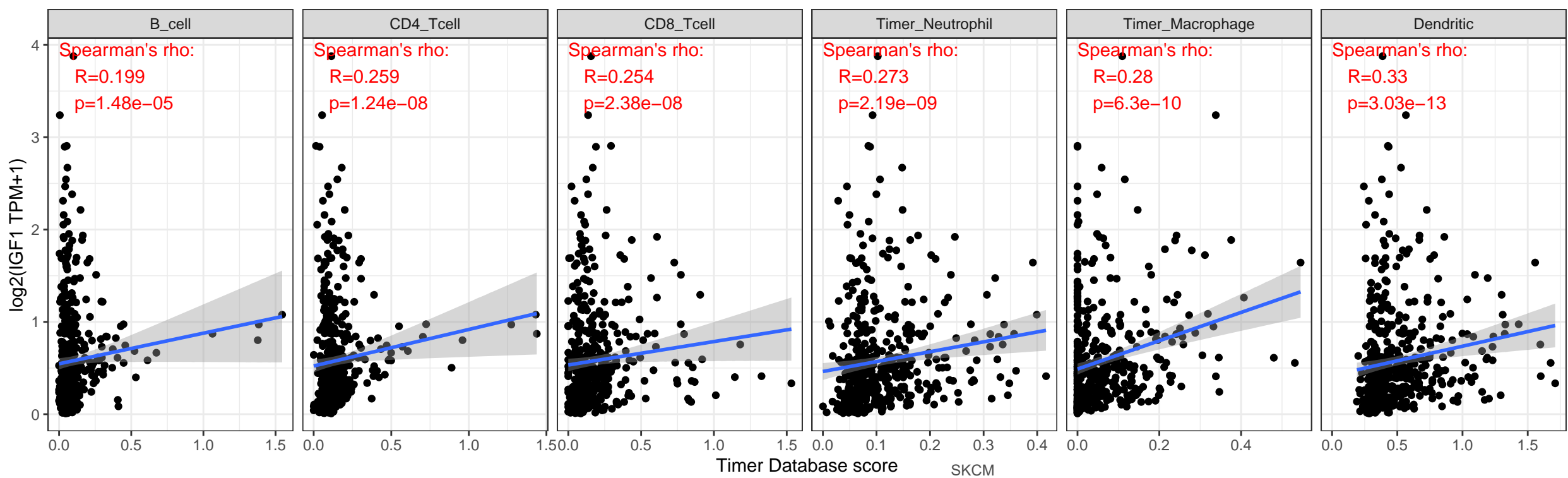

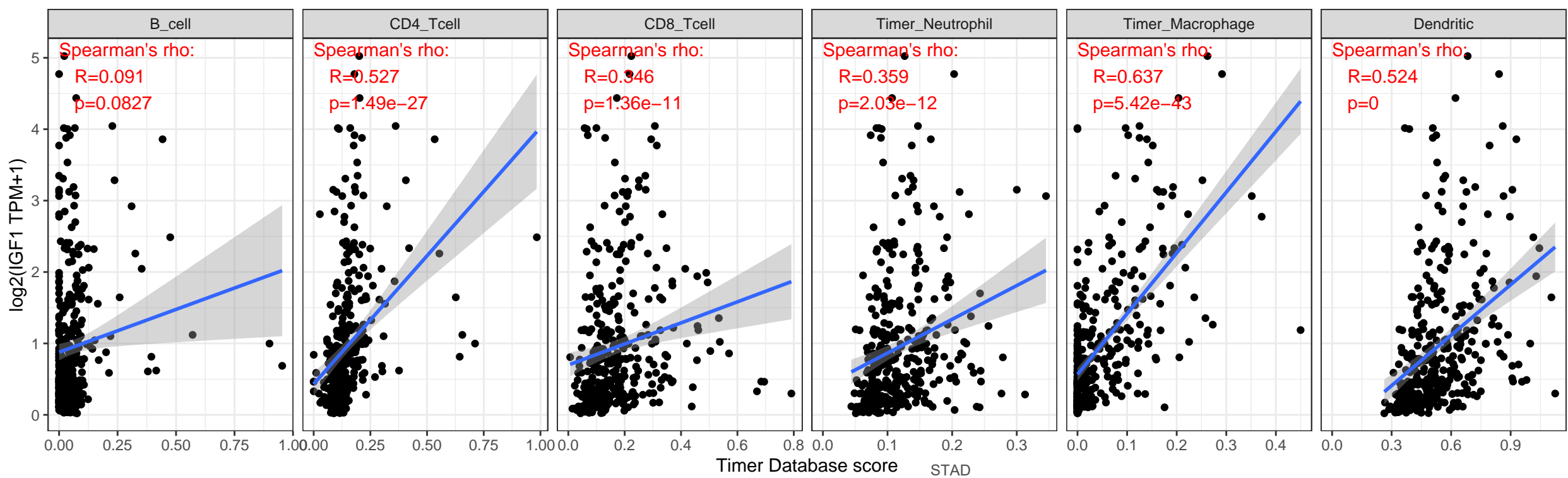

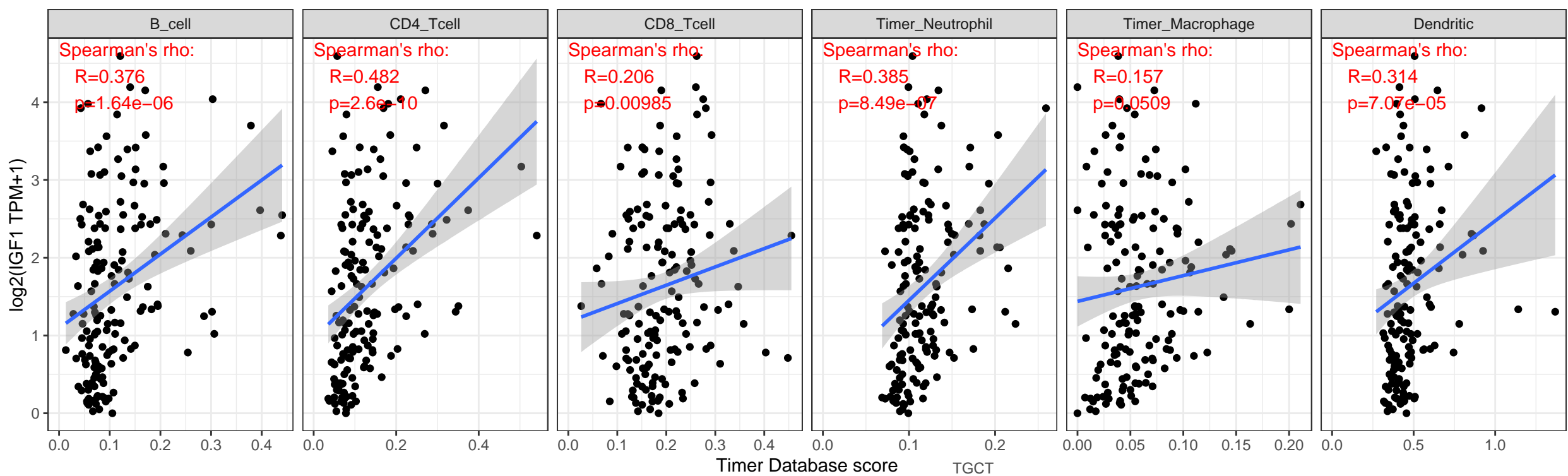

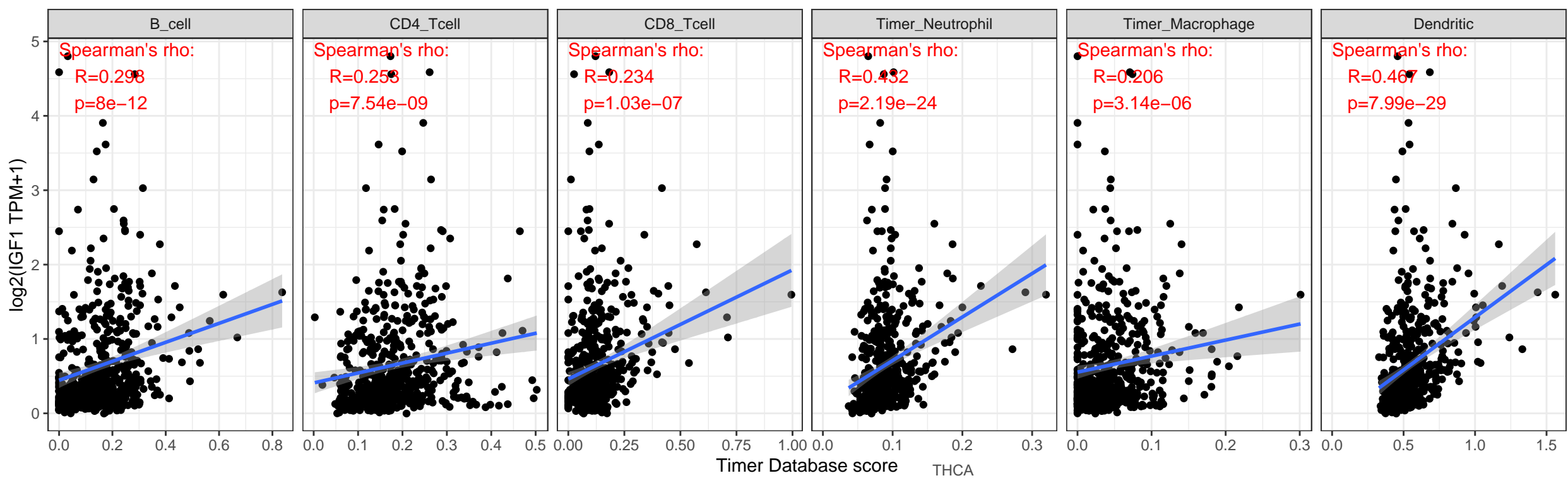

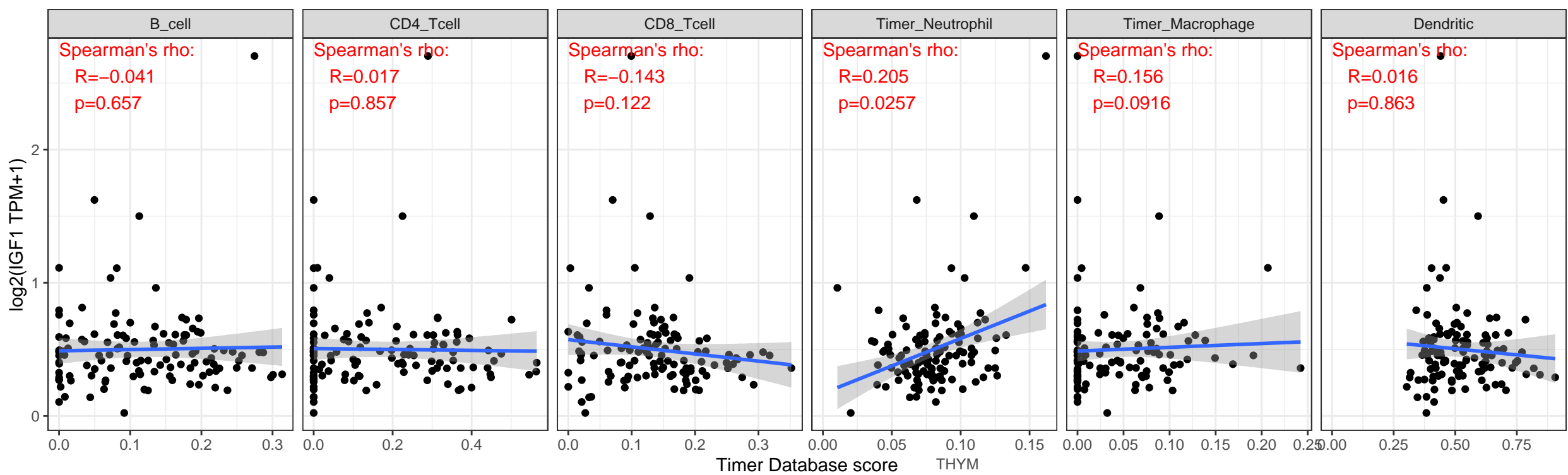

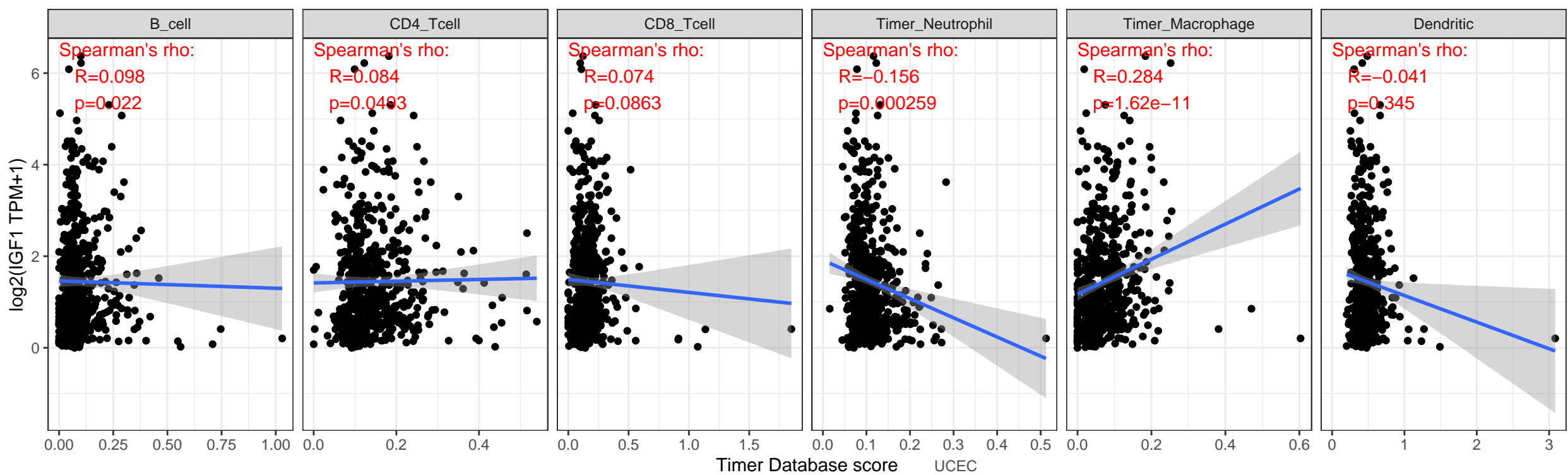

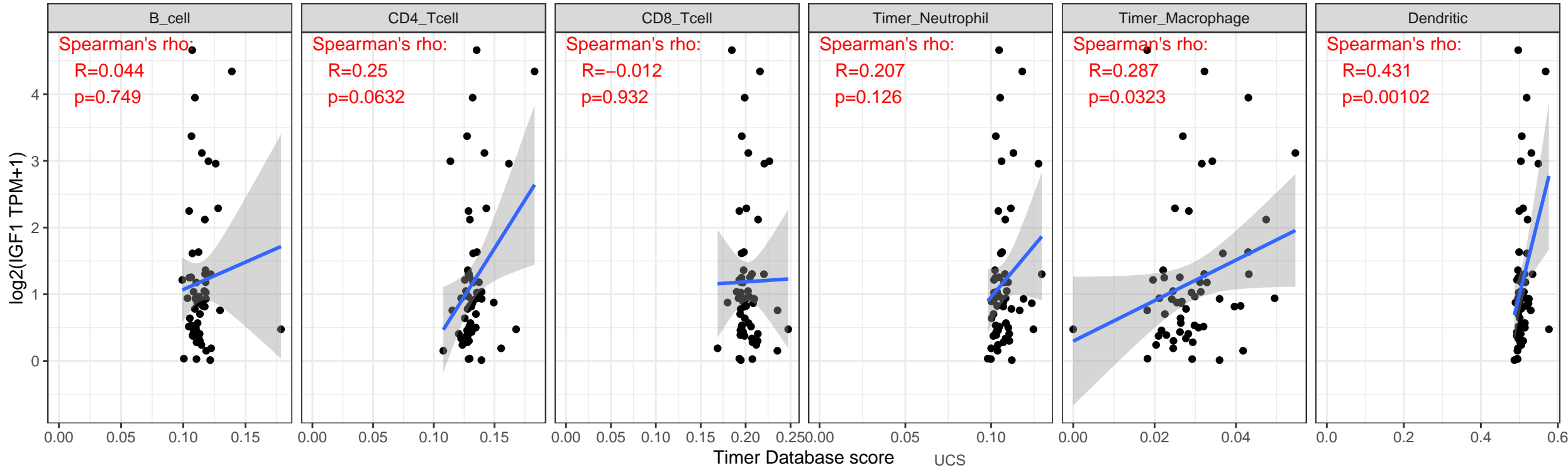

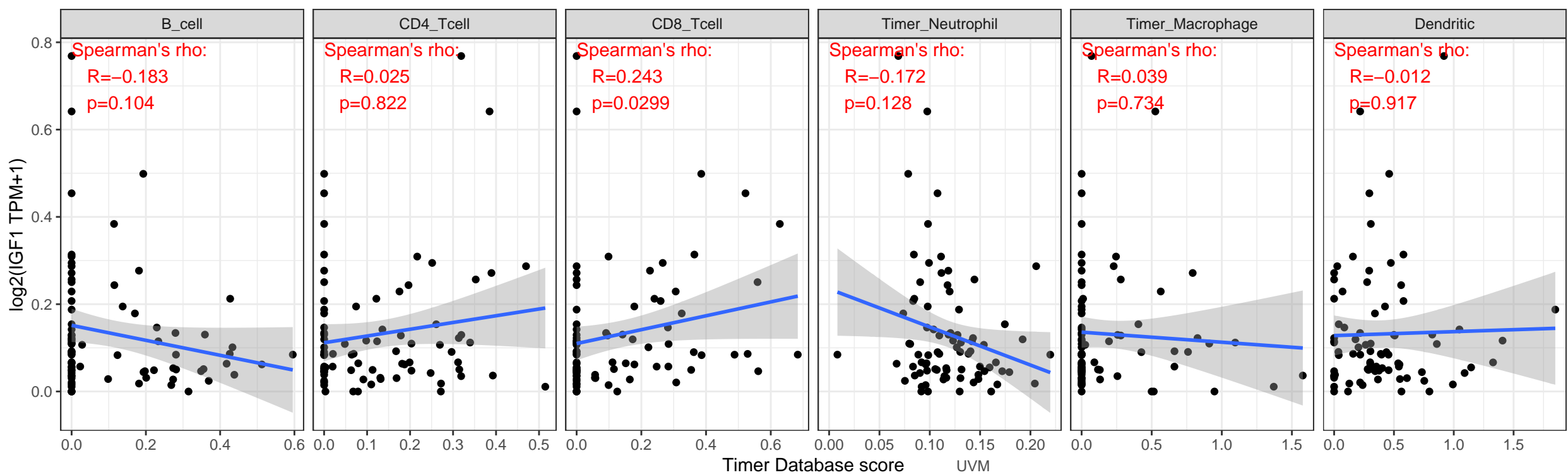

Supplement: Supplementary file 7 [file DataSheet_3.pdf]

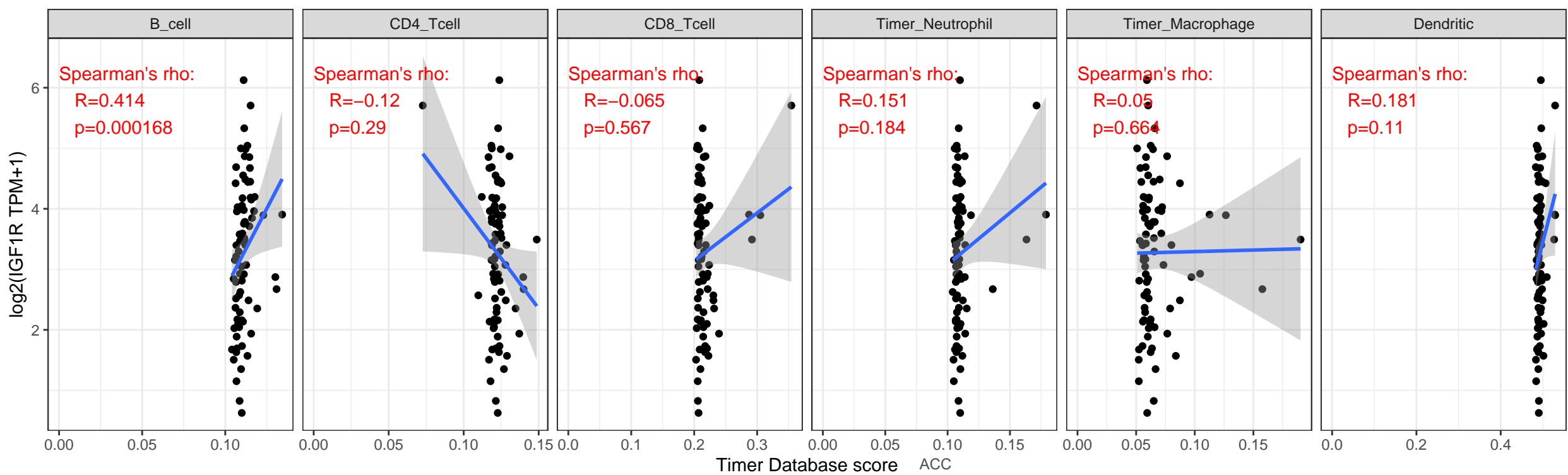

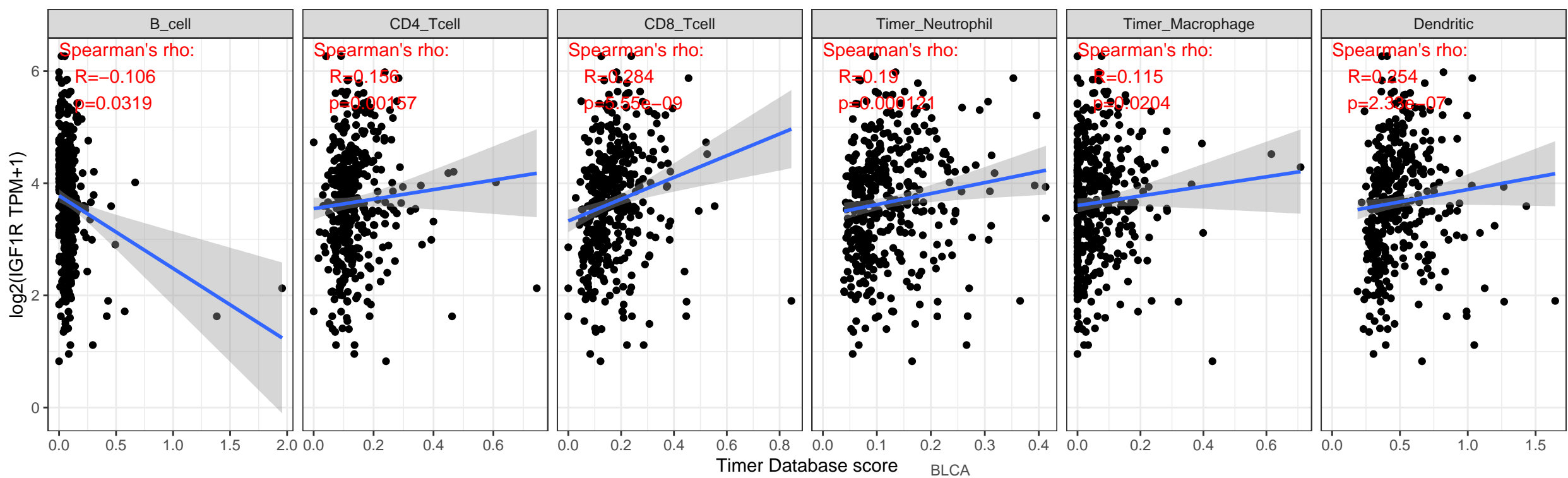

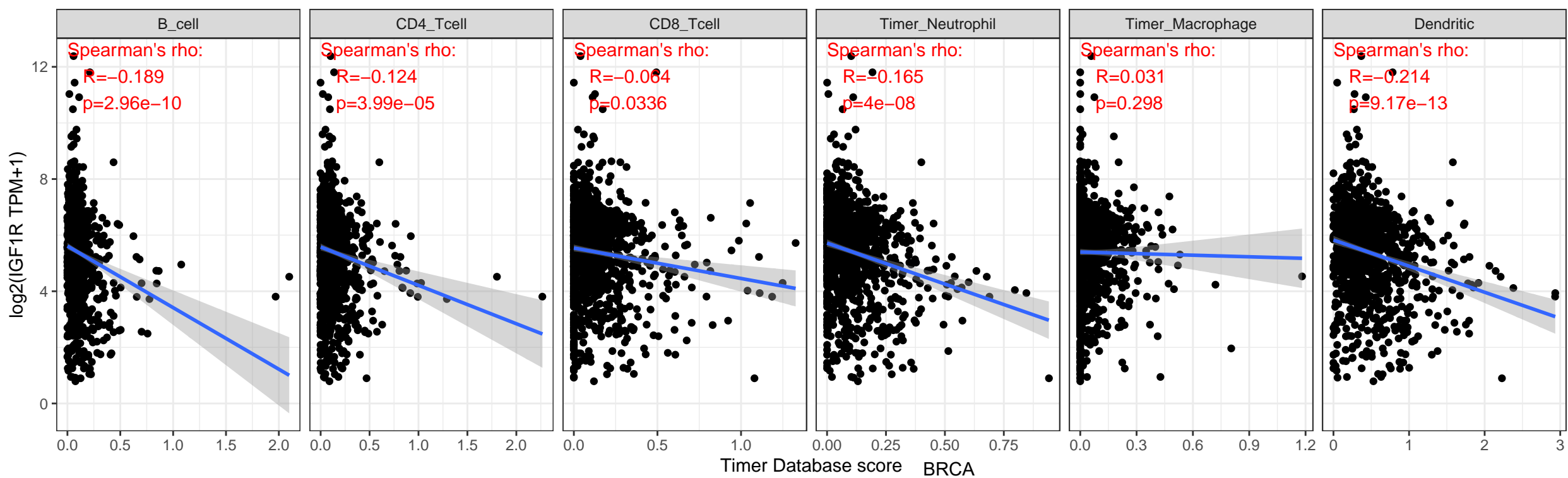

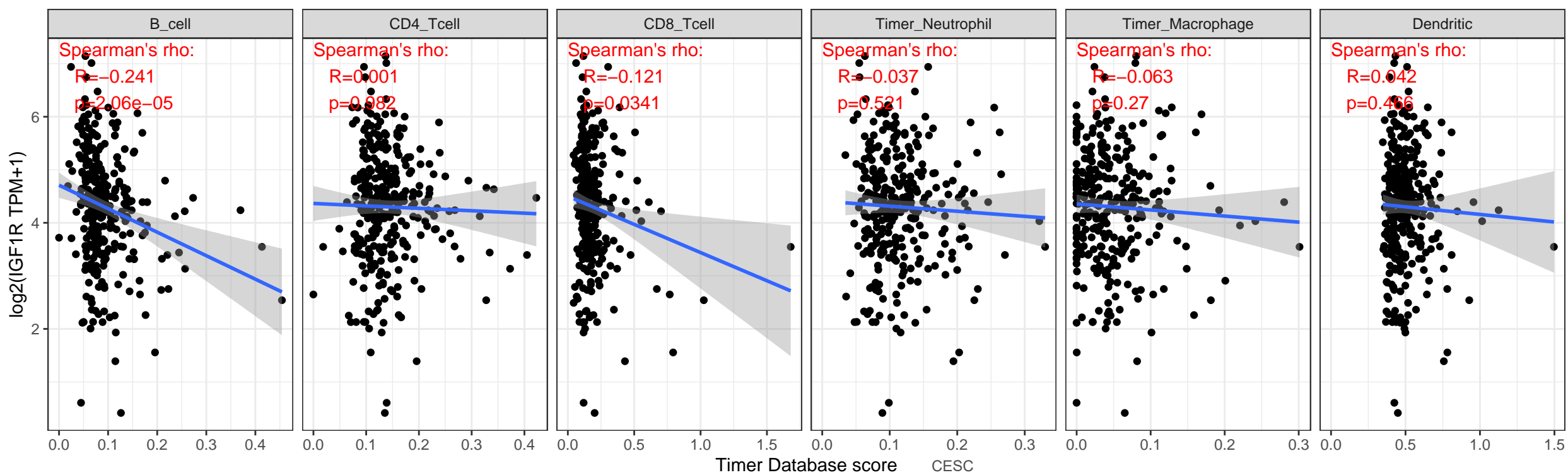

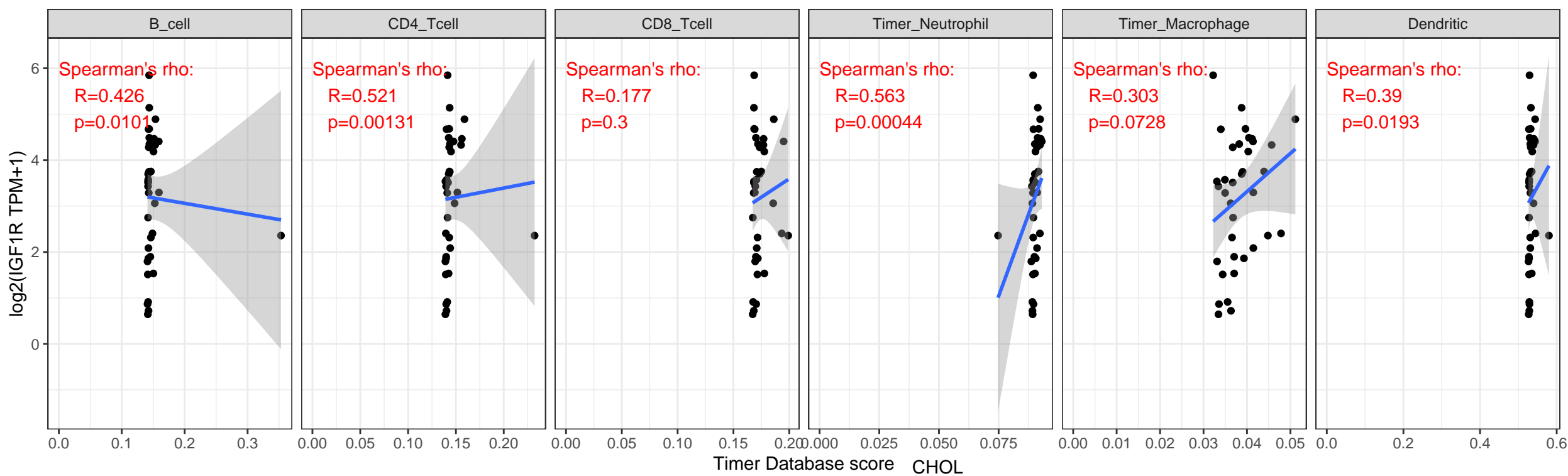

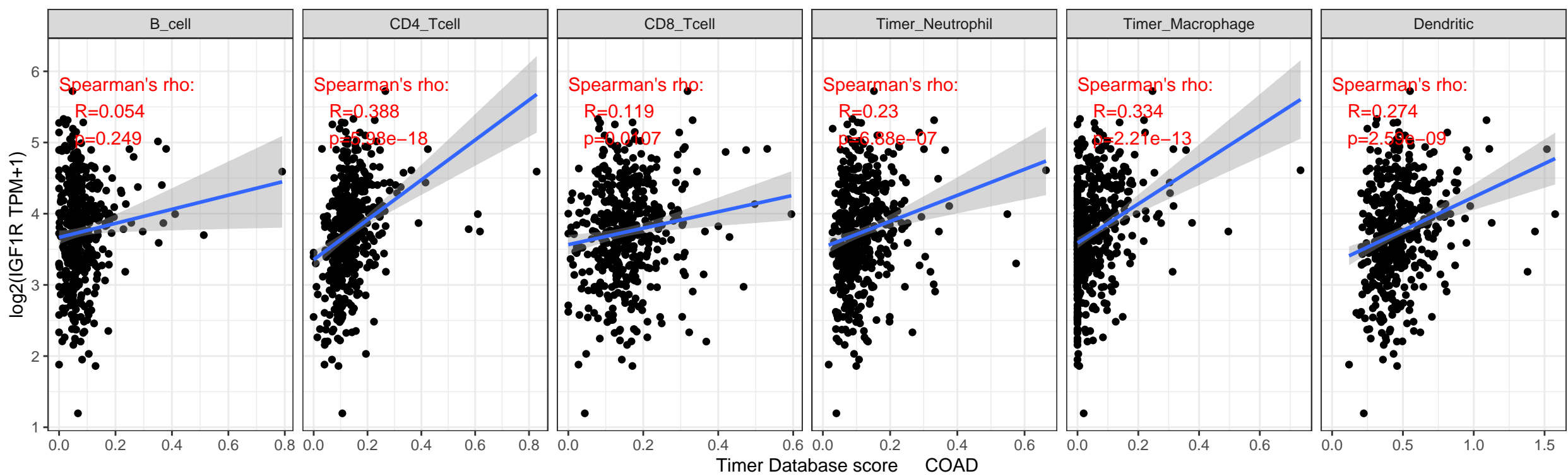

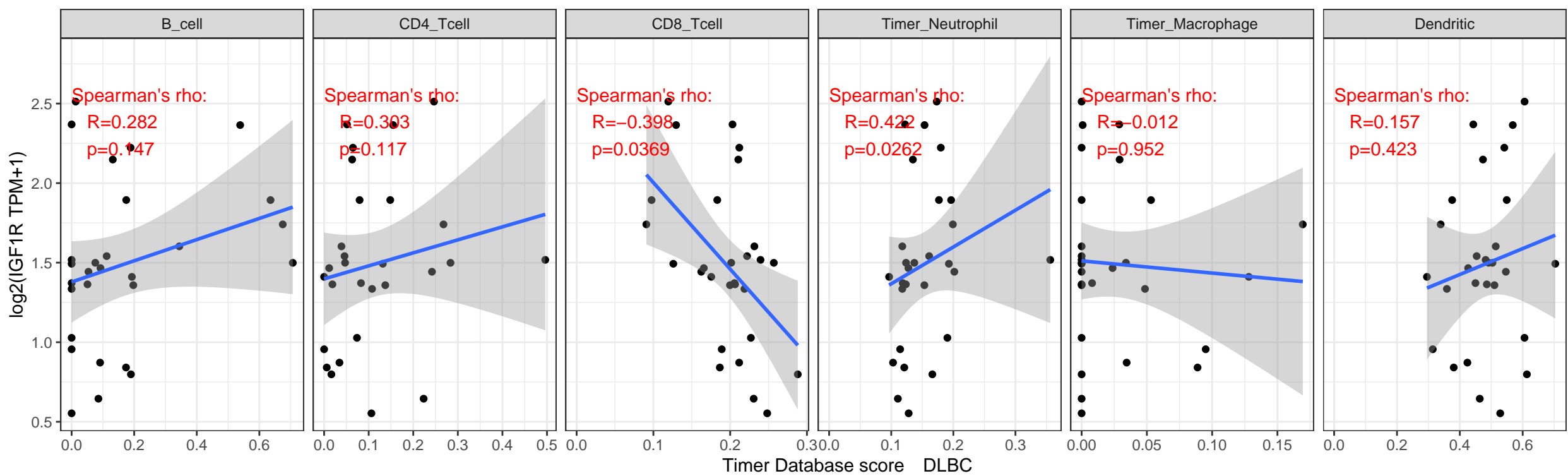

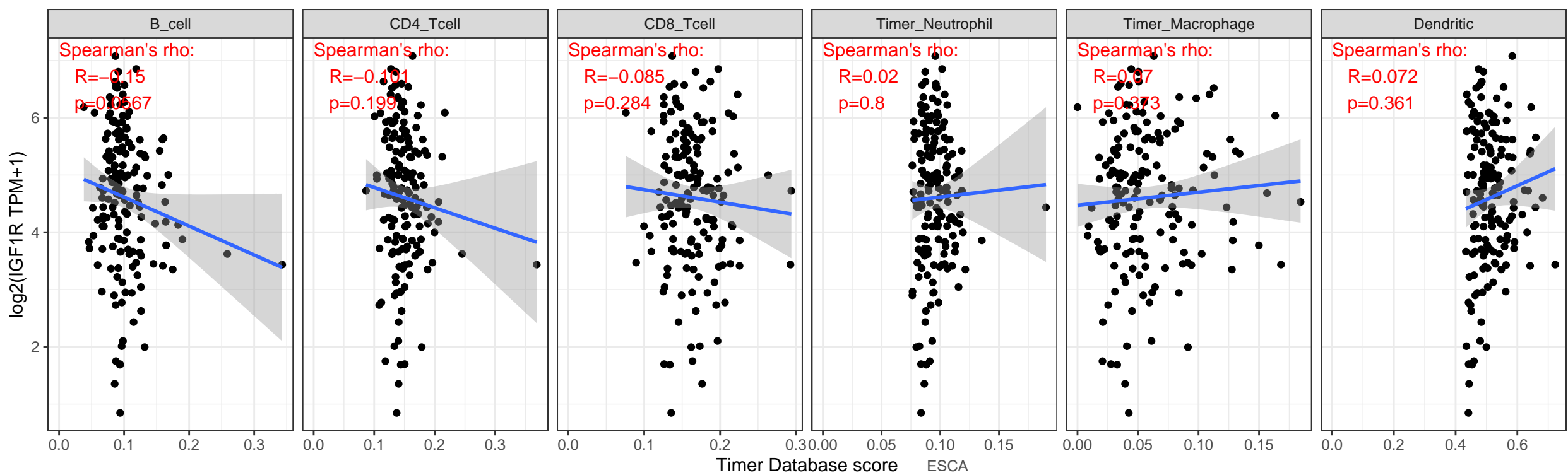

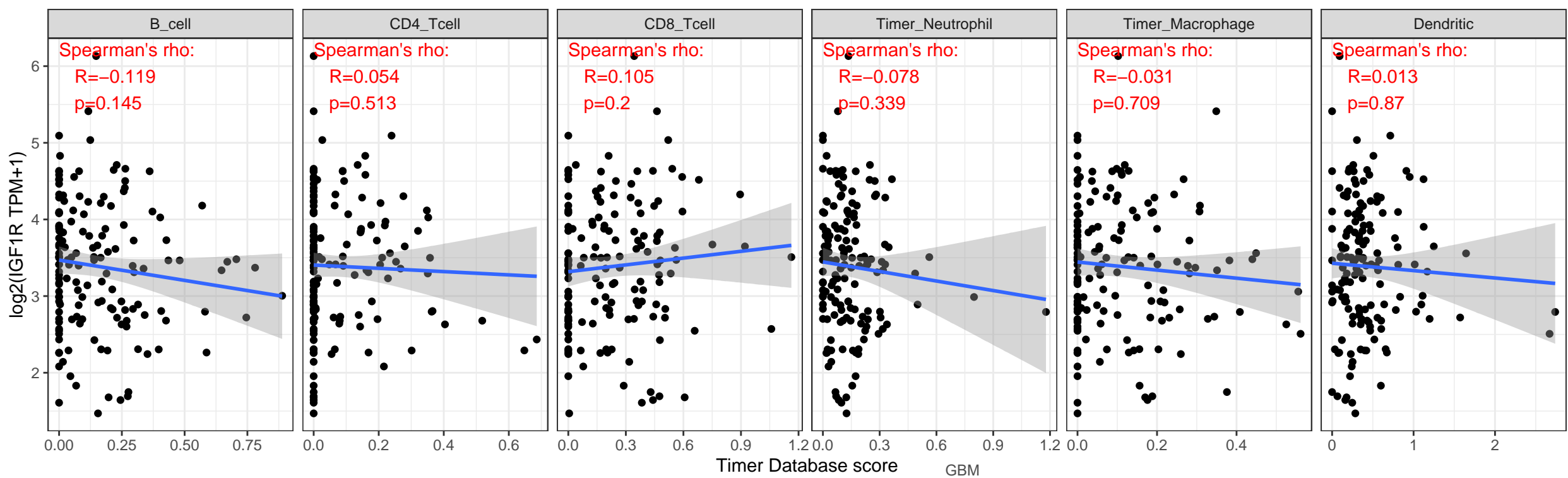

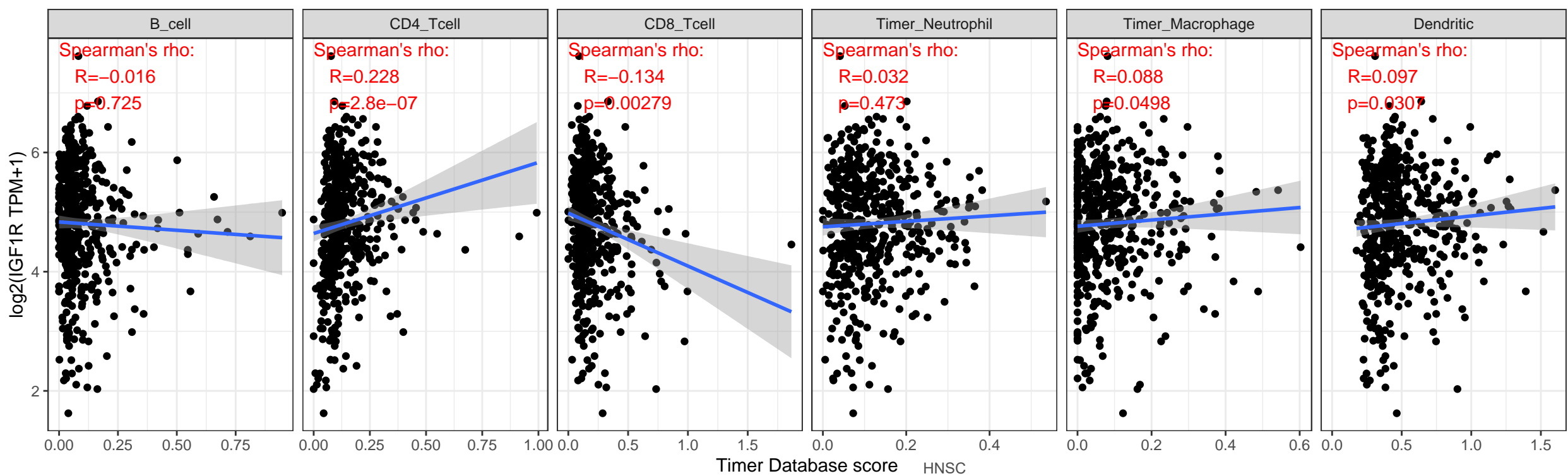

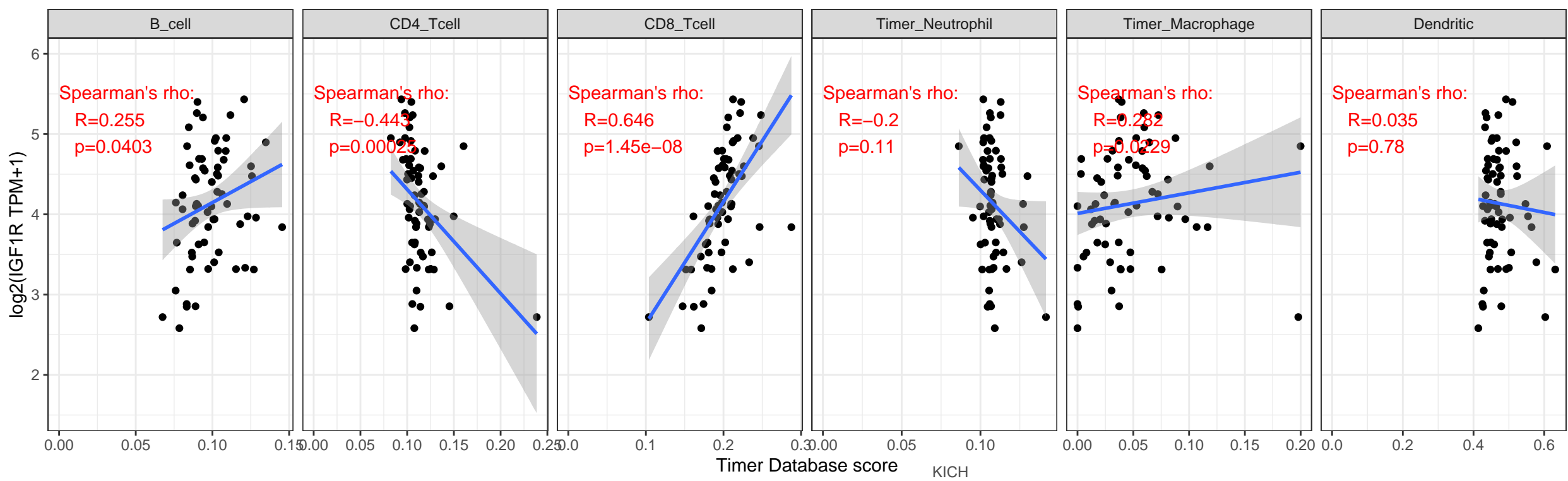

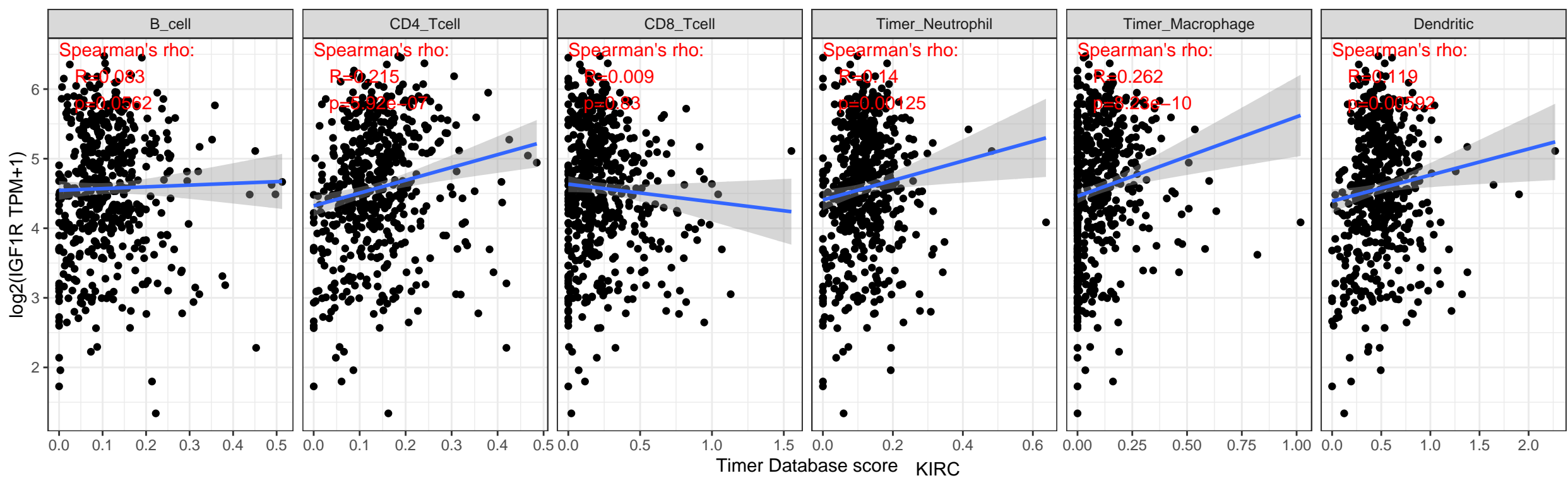

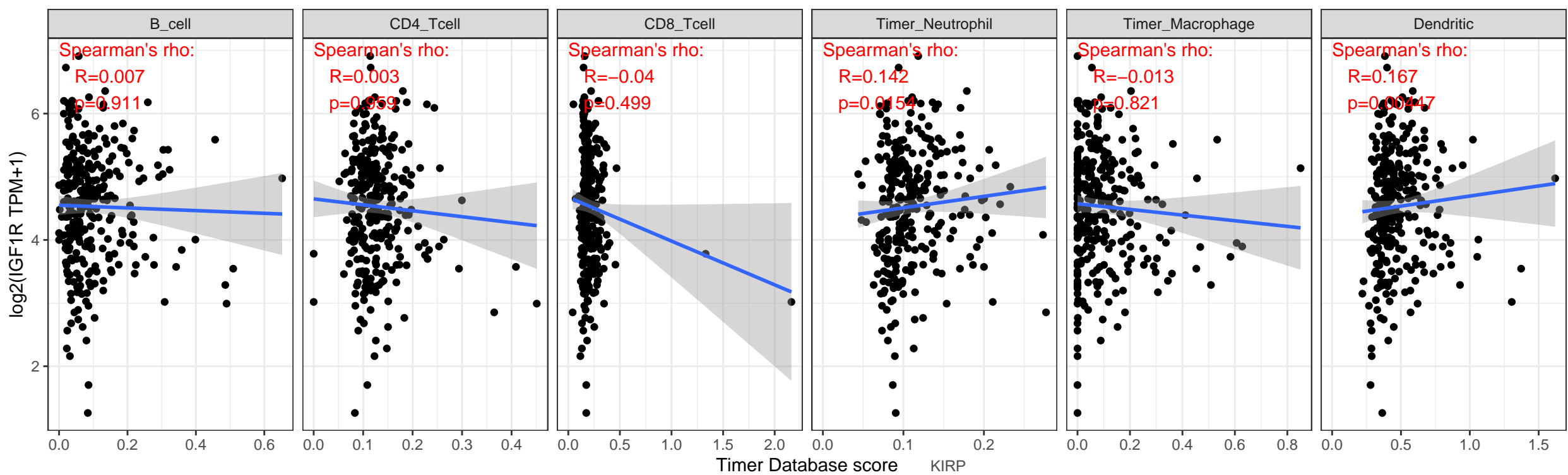

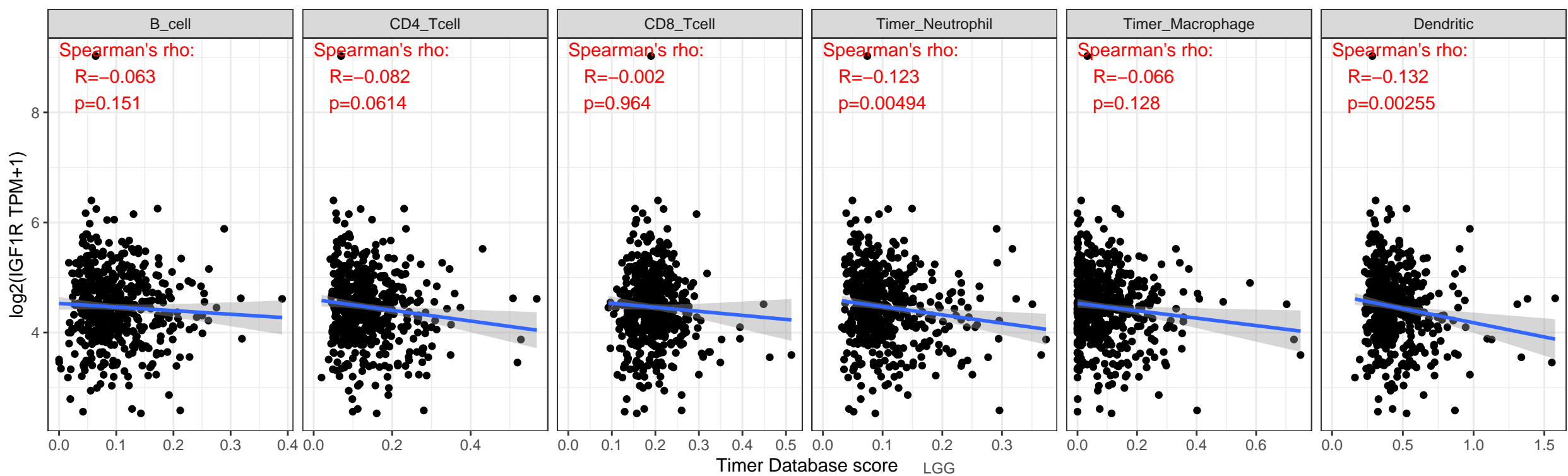

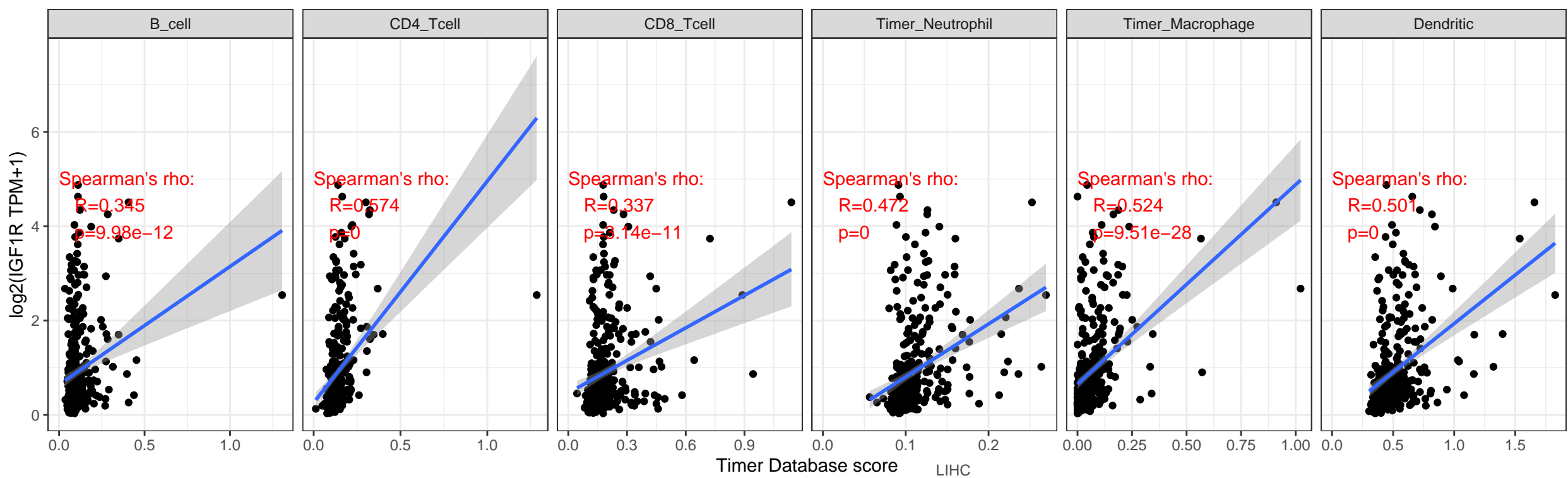

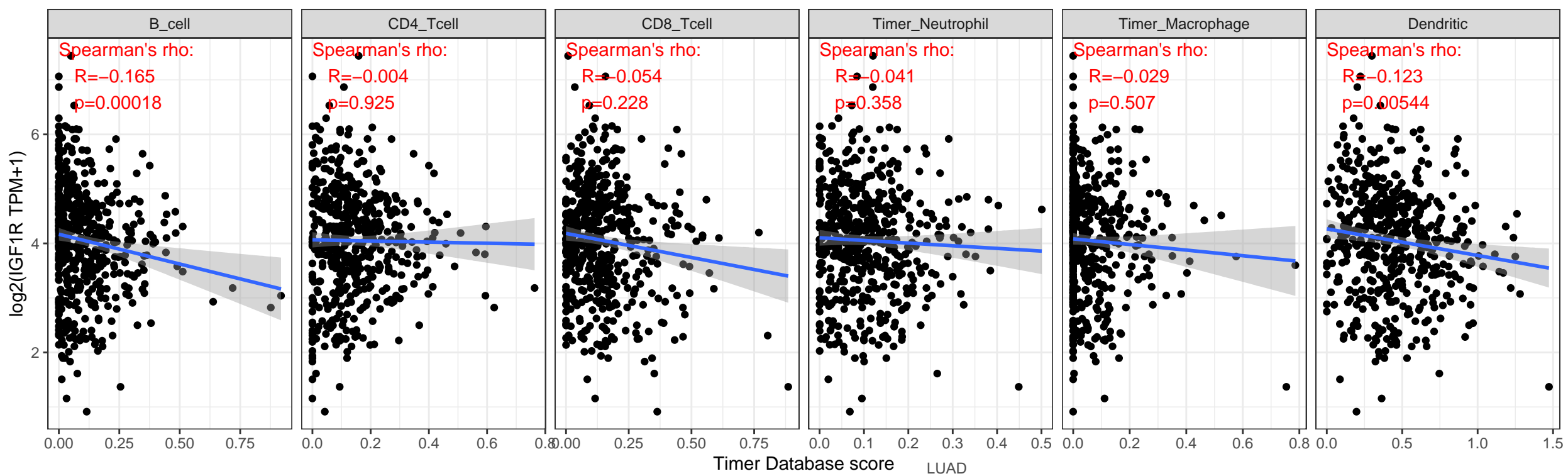

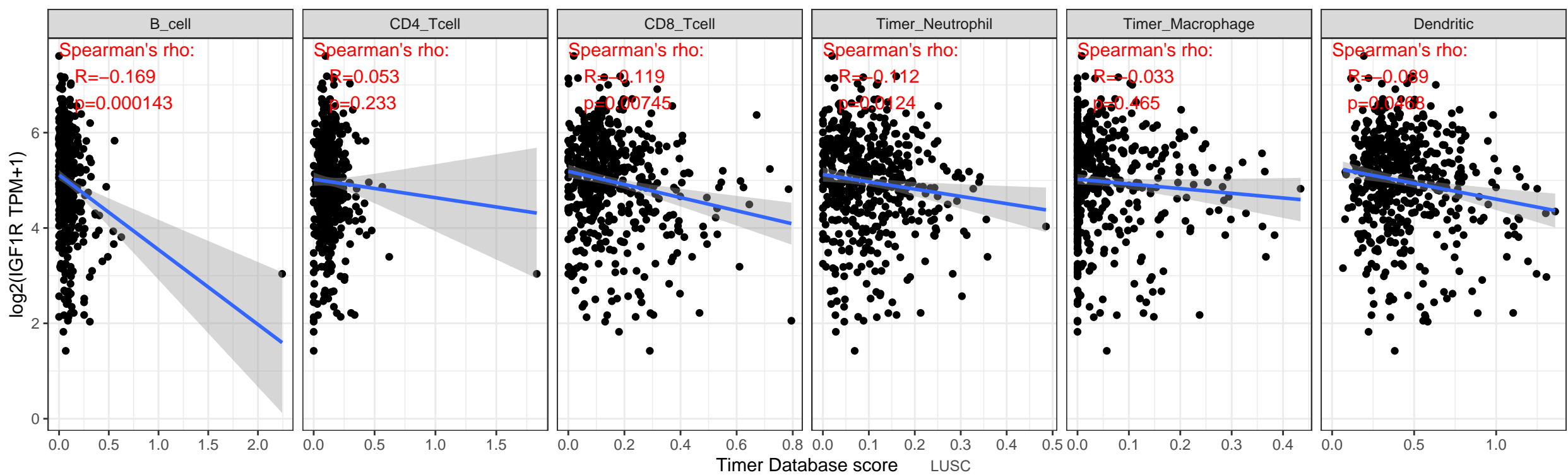

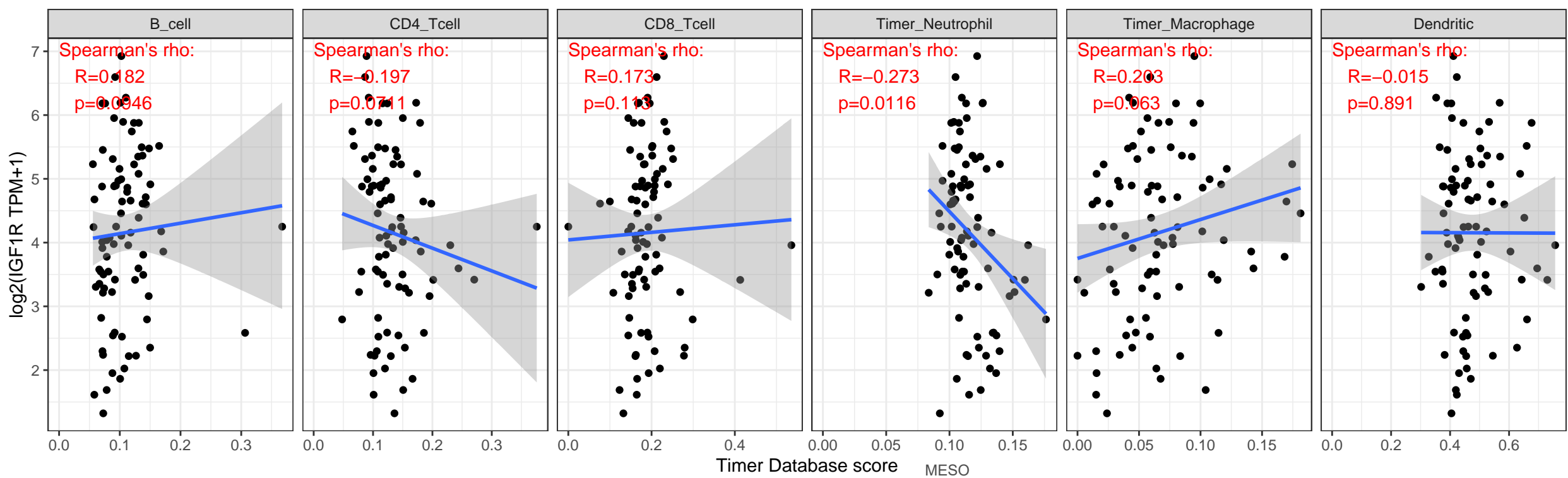

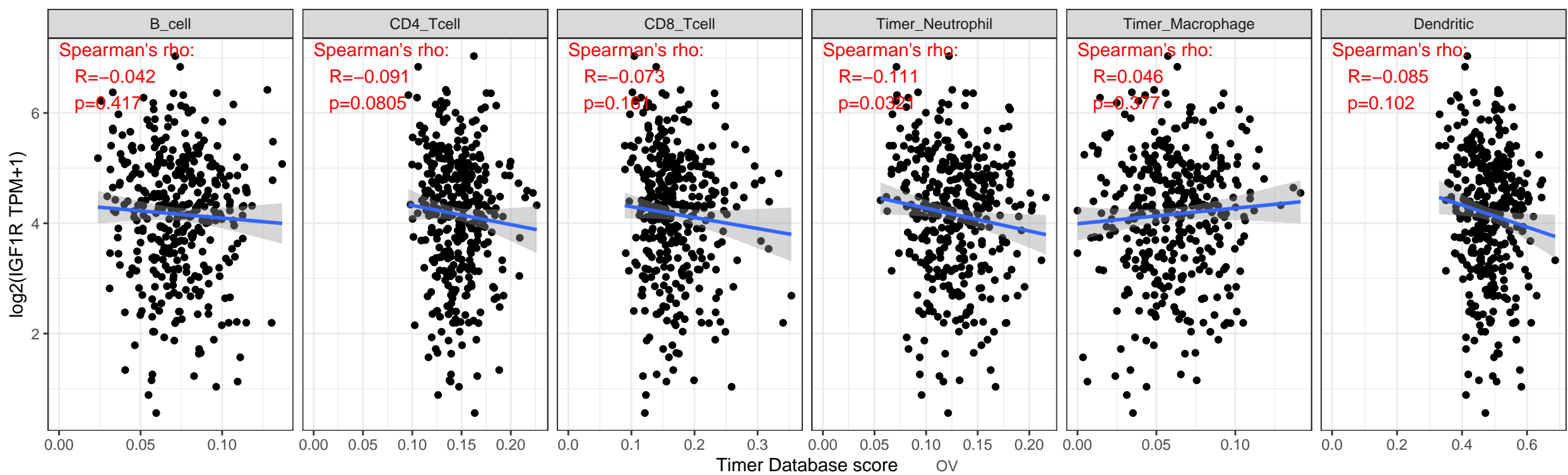

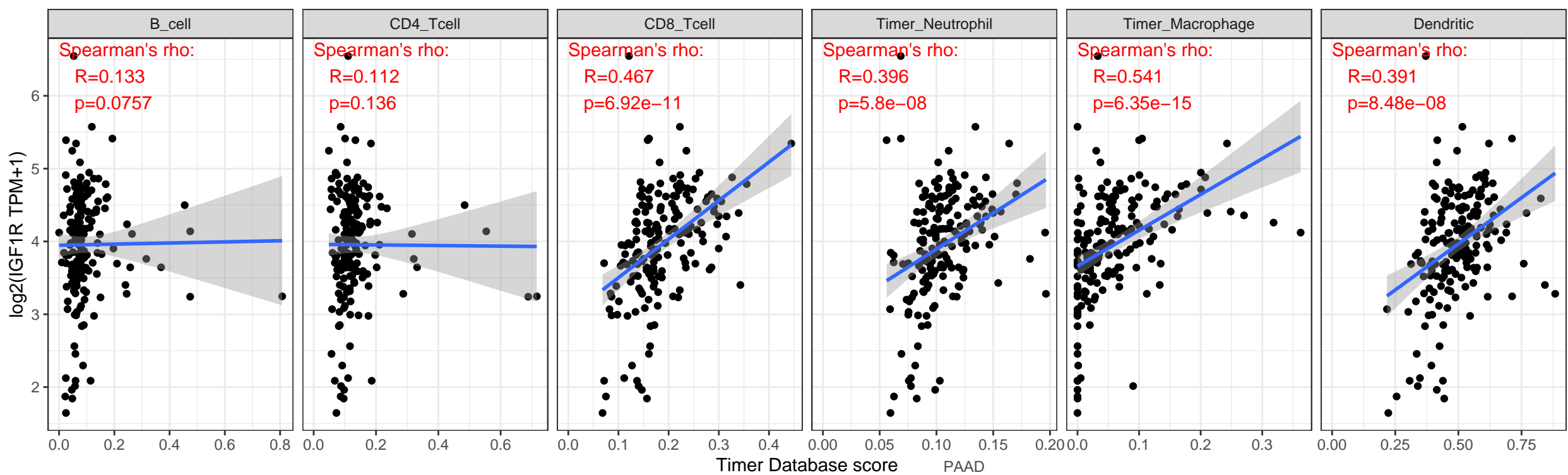

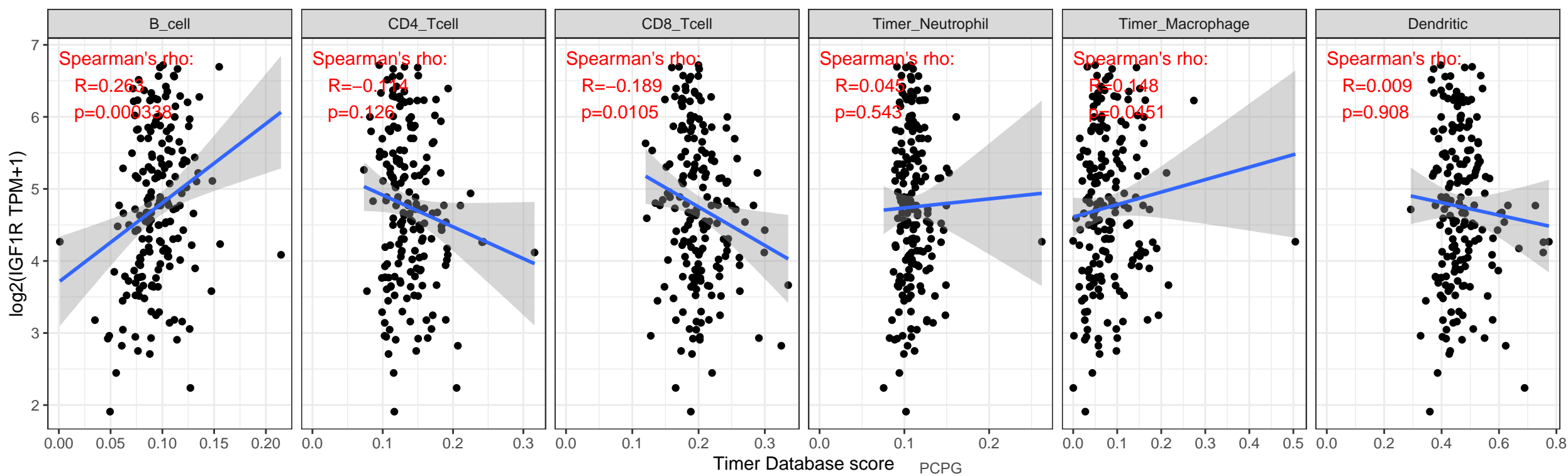

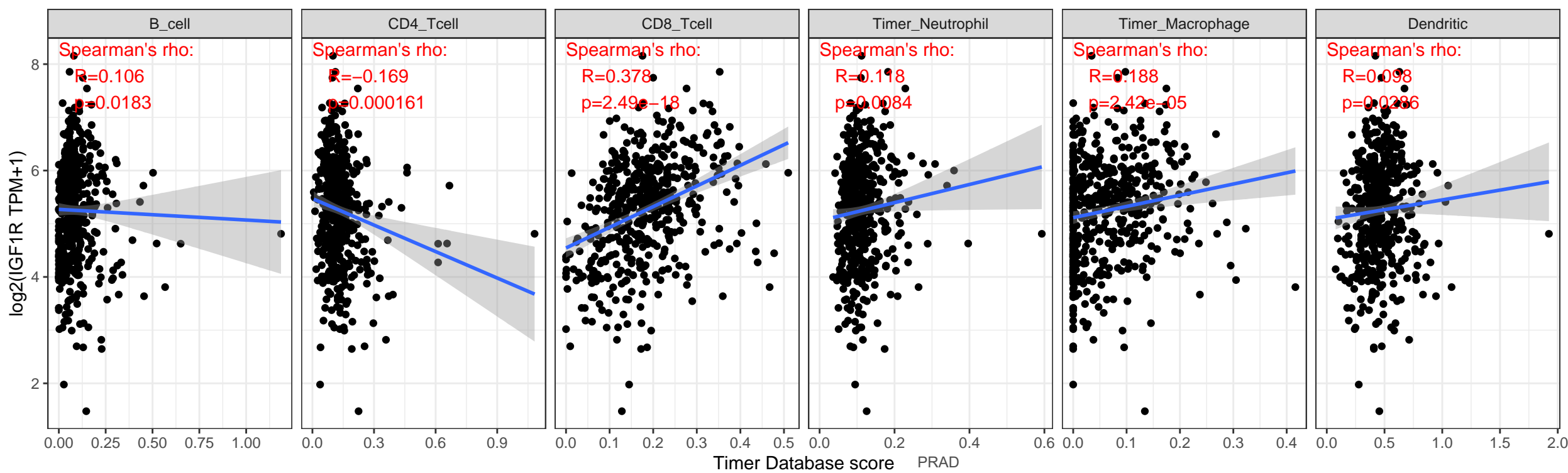

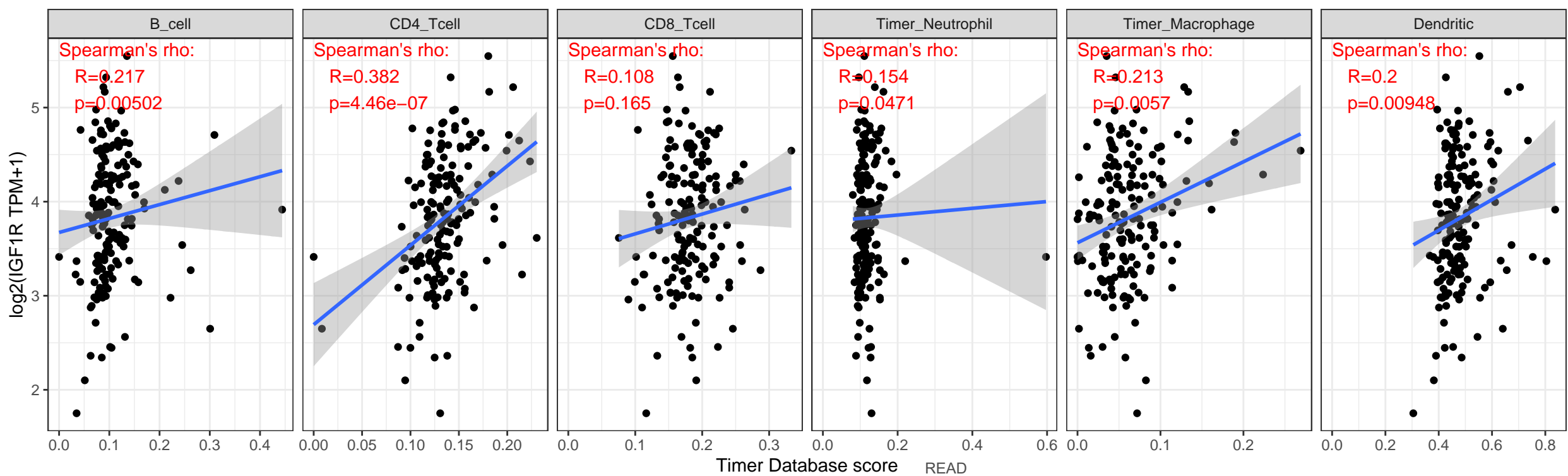

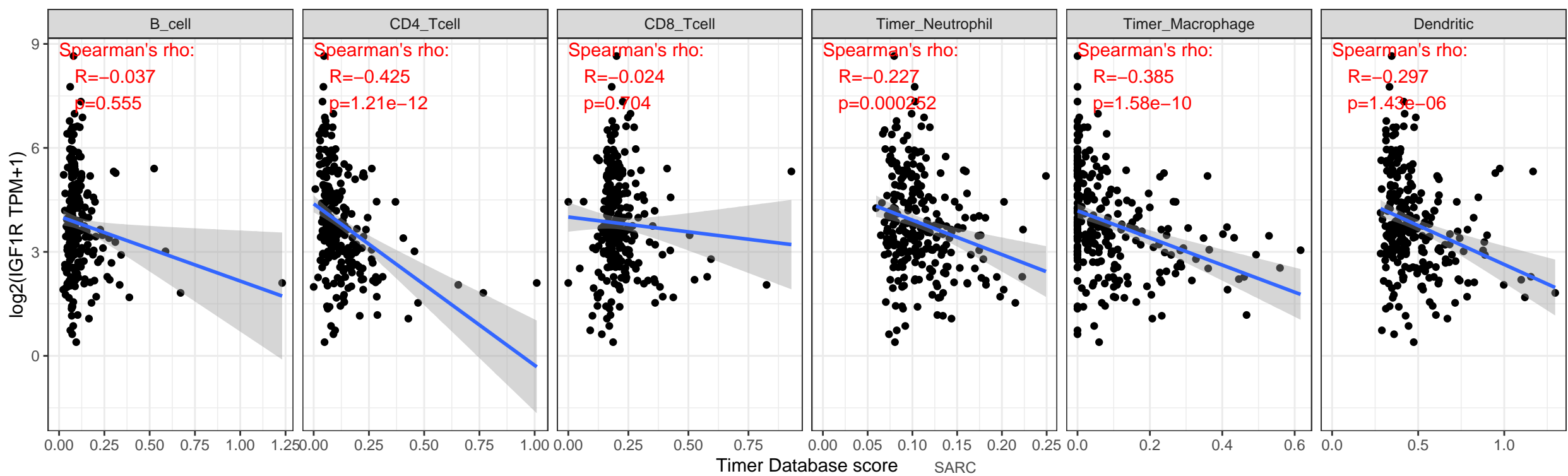

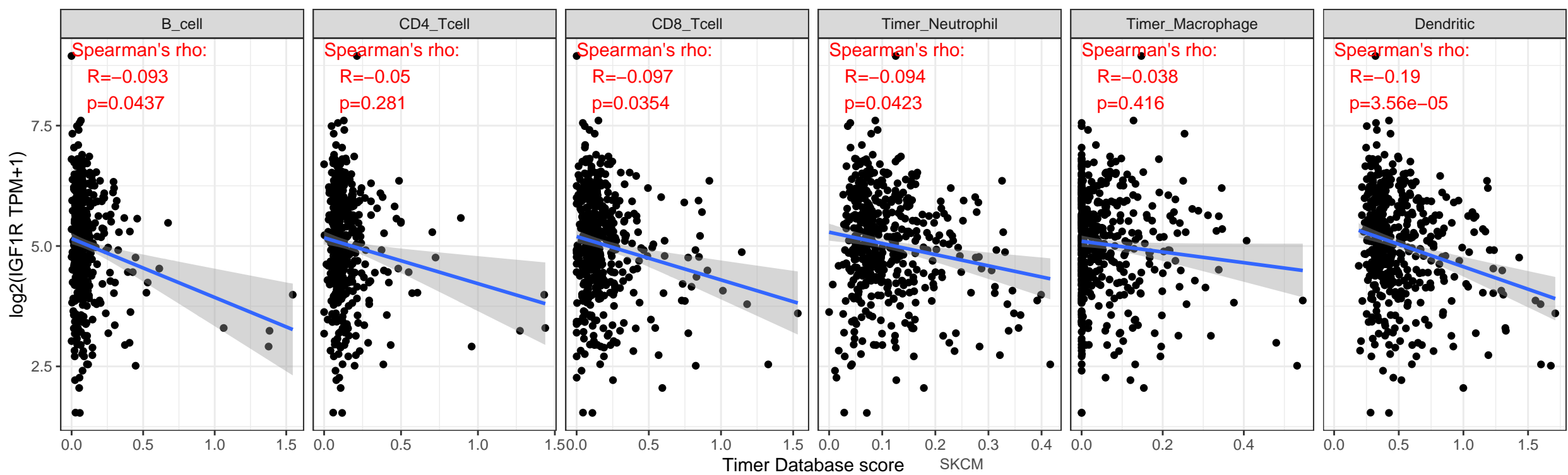

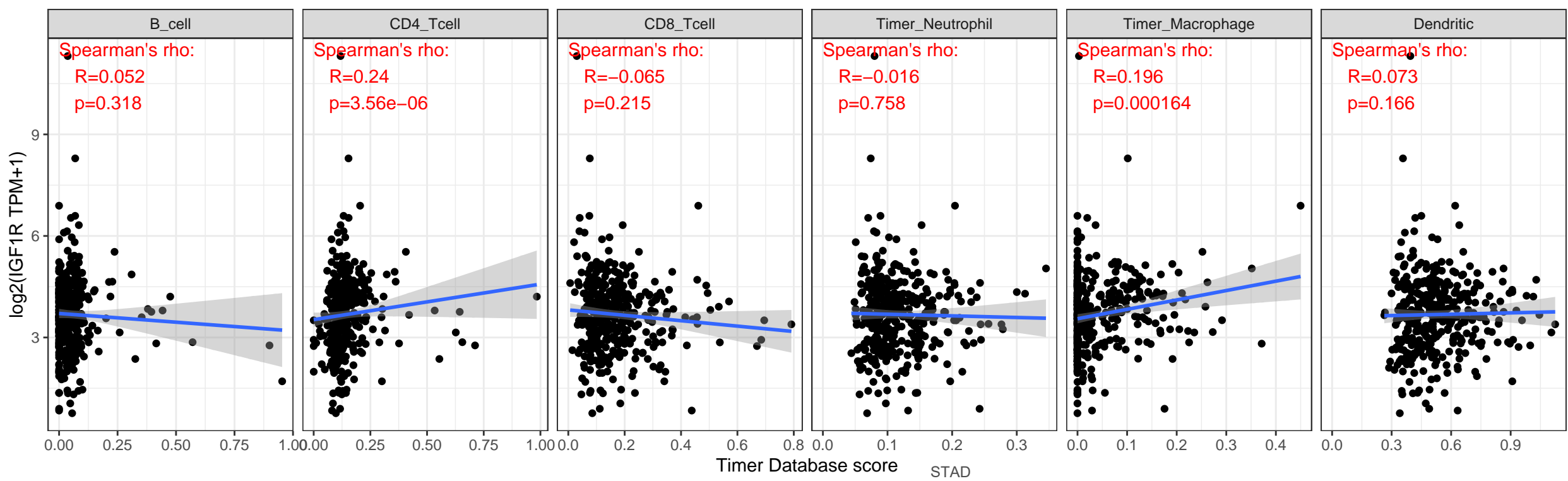

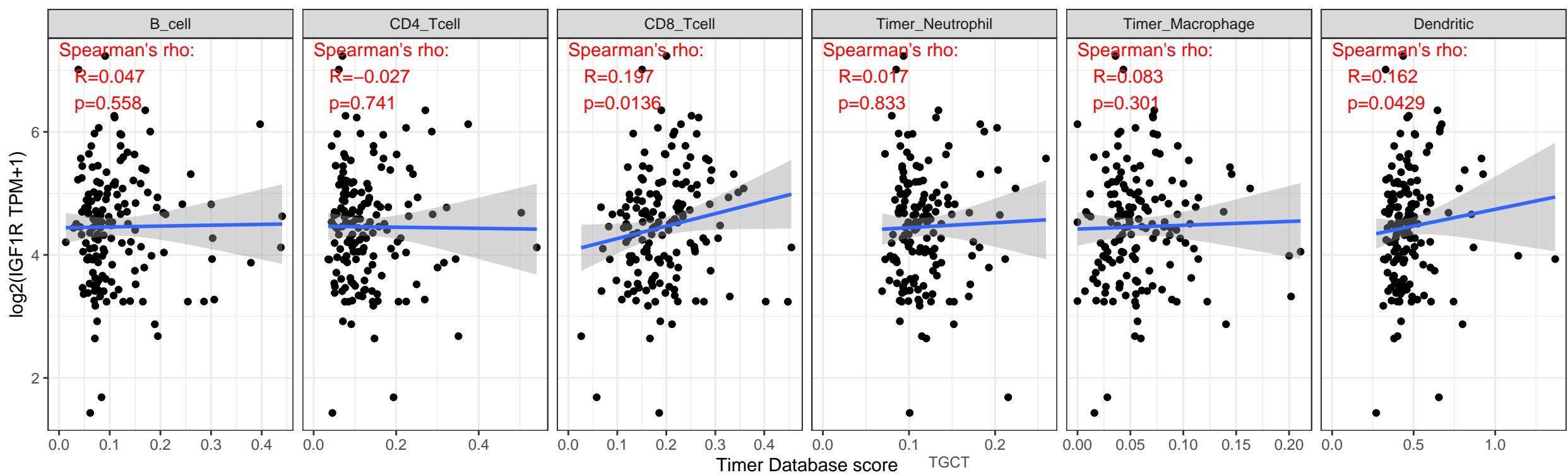

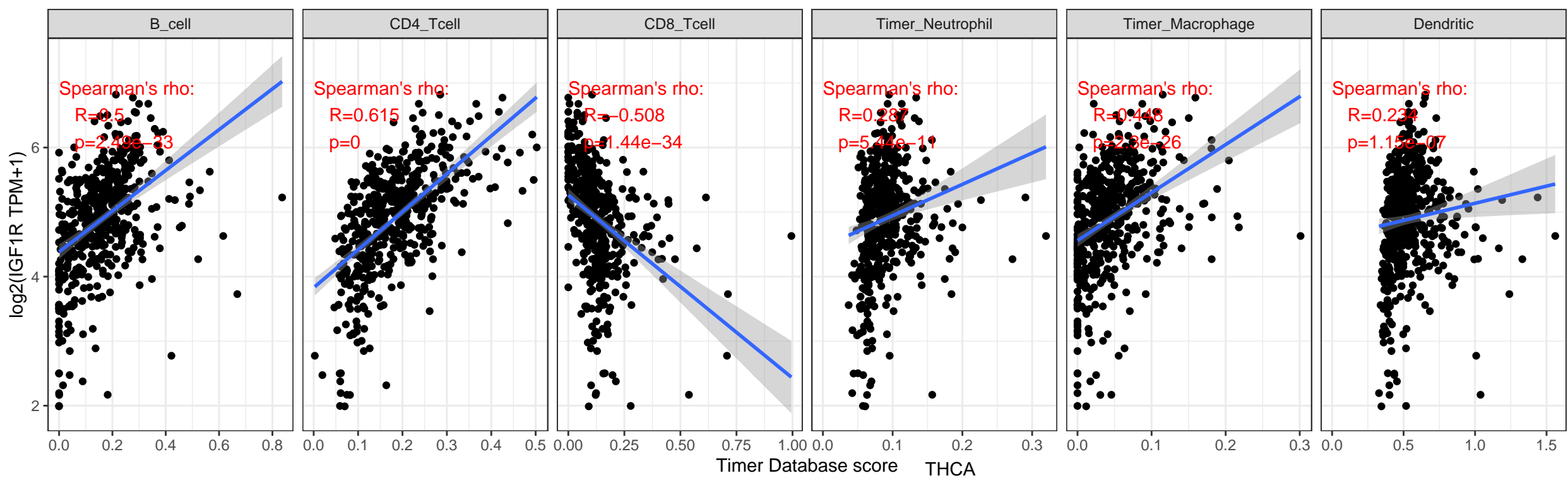

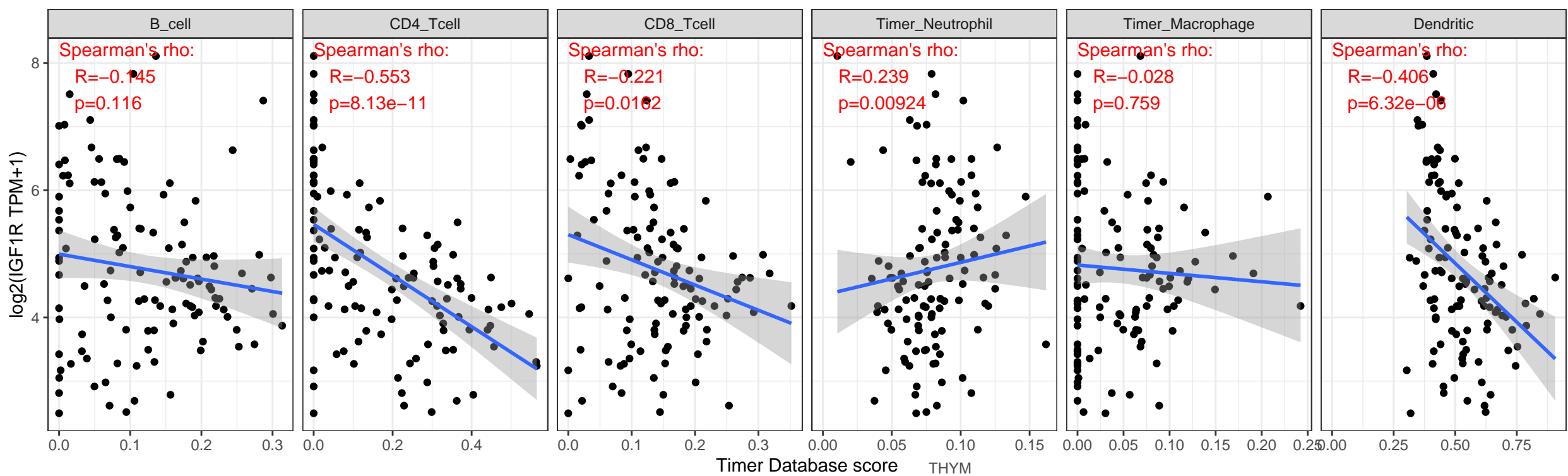

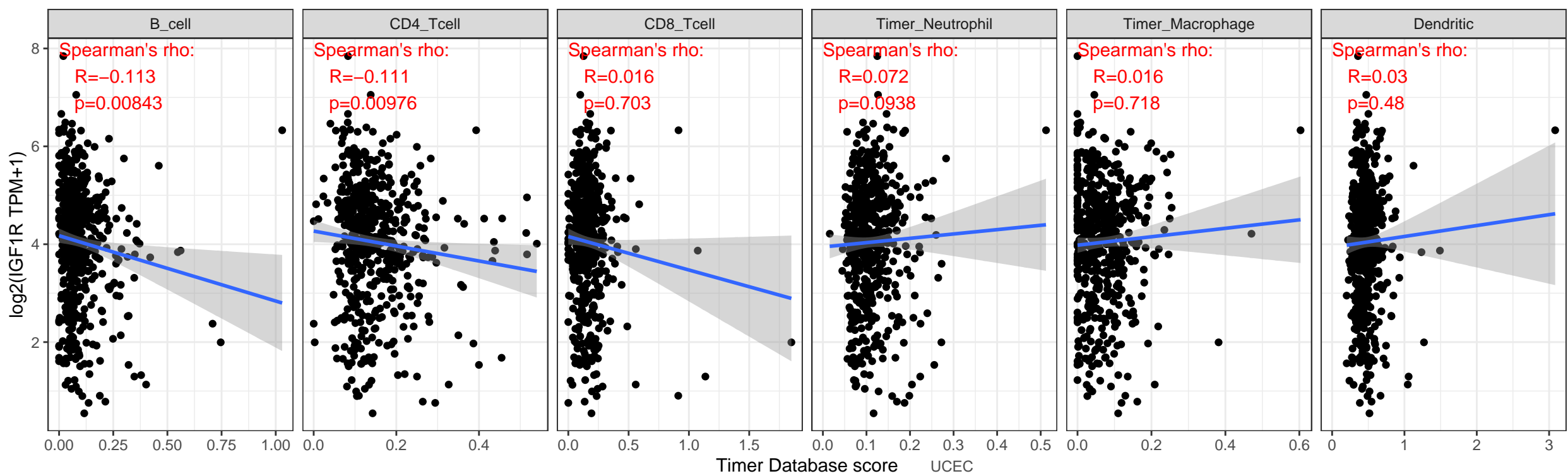

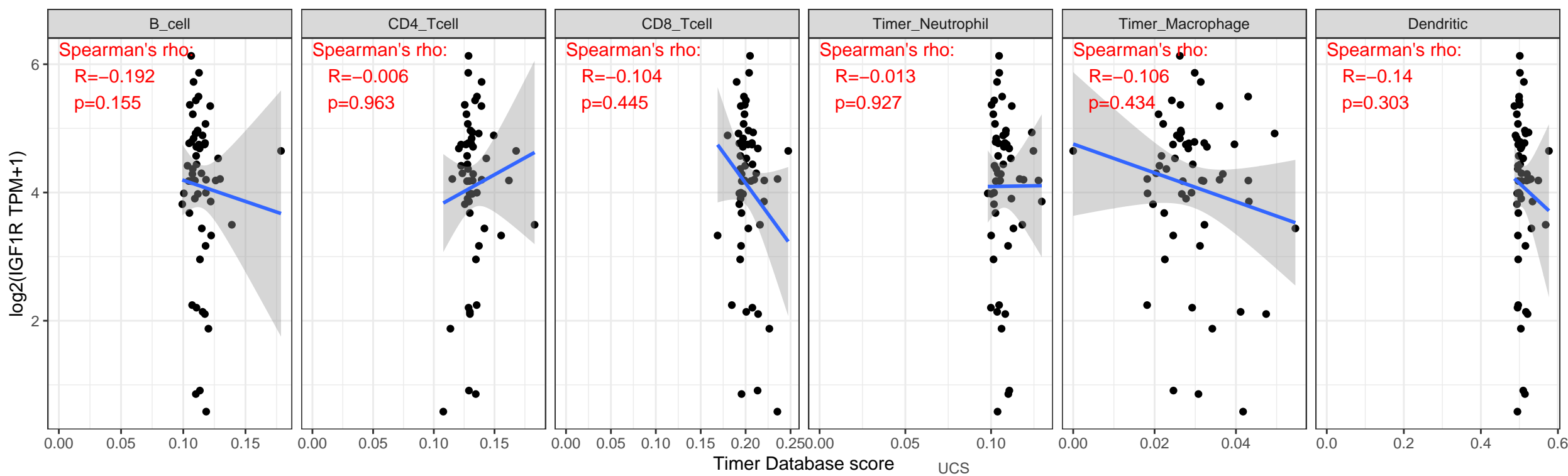

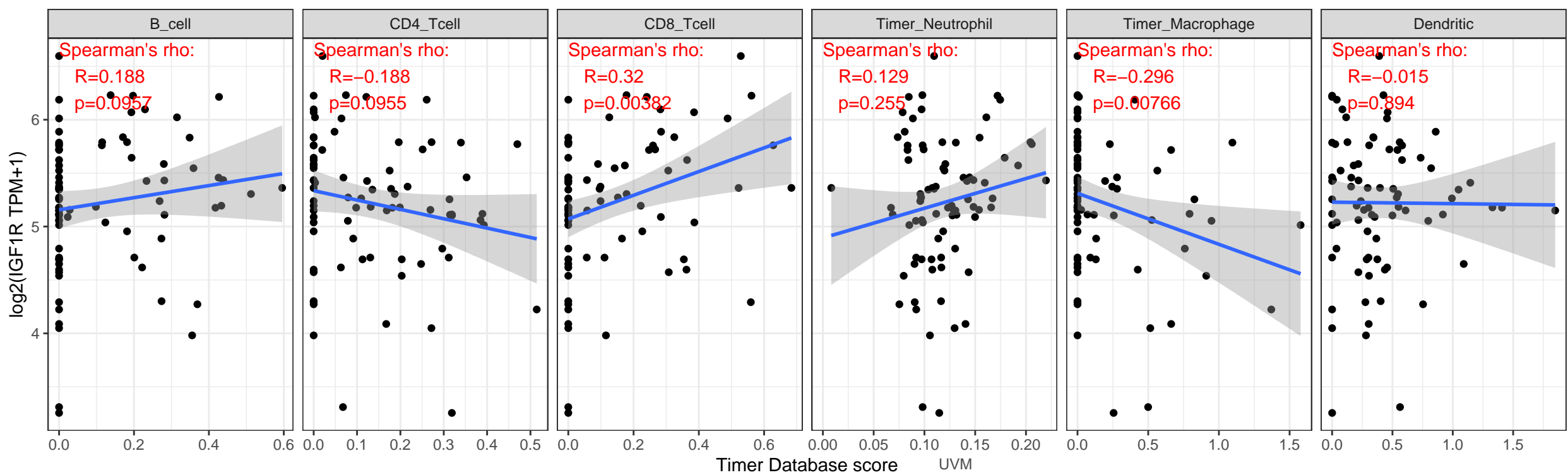

Supplement: Supplementary file 8 [file DataSheet_4.pdf]
